# Preparation, characterization and photocatalytic performance of heterostructured CuO–ZnO-loaded composite nanofiber membranes

**DOI:** 10.3762/bjnano.11.50

**Published:** 2020-04-15

**Authors:** Wei Fang, Liang Yu, Lan Xu

**Affiliations:** 1 National Engineering Laboratory for Modern Silk, College of Textile and Engineering, Soochow University, 199 Ren-ai Road, Suzhou 215123, China

**Keywords:** electrospinning, composite nanofibers, heterostructured CuO–ZnO, hydrothermal synthesis, photocatalysis, semiconductor oxide

## Abstract

Inorganic semiconductor oxides loaded on composite nanofibers (CNFs) have been widely applied in environmental monitoring, industry, aviation, and transportation. In this paper, heterostructured CuO–ZnO-loaded CNF membranes (CNFMs) were prepared successfully by a combination of electrospinning, heat treatment, and hydrothermal synthesis. The influence of the synthesis parameters on morphology, structure, and properties of the CNFMs was investigated, and the optimal process parameters were determined. Then, the CNFMs obtained with optimal process parameters were applied for the photocatalytic degradation of methyl orange. It was found that the CNFMs could be reused to degrade methyl orange at least three times, and the degradation rate remained above 90%.

## Introduction

Water remediation is one of the main scientific research subjects regarding environmental protection. Water pollution with organic dyes (such as congo red, methylene blue, and methyl orange) is becoming a major environmental problem. Therefore, water purification technologies, such as photocatalytic purification, electrochemical oxidation, membrane filtration, ozonation, and chlorination flocculation, have attracted much attention recently [1–3]. The photocatalytic purification of water has the advantages of high efficiency, thoroughness, and no secondary pollution. Various refractory organic pollutants in water are thoroughly oxidized to non-toxic and less harmful substances. Thus, photocatalytic purification might become the main means for the treatment of water and air pollutants [4].

Photocatalytic reactions on metal oxide semiconductors can degrade many pollutants and are, thus, of great interest [5]. Photogenerated charge carriers formed through bandgap excitation can reduce or oxidize species adsorbed on the semiconductor material. However, the high degree of recombination of charge carriers is disadvantage [6]. The coupling of two different semiconductors can yield an efficient charge separation, leading to a vector transmission of photogenerated electrons and holes from one semiconductor material to the other [7–10]. ZnO is a semiconductor material with a wide direct bandgap of 3.2 eV, which can absorb a small part of the solar spectrum in the UV region [11–13]. CuO is a nontoxic, chemically stable and naturally abundant material. It is a p-type semiconductor with a narrow direct bandgap of 1.2–1.79 eV [14]. Because of that, CuO is usually used in combination with large-bandgap semiconductors, such as ZnO and TiO_2_, in order to improve their photocatalytic activity under solar light irradiation [15]. It was reported that the p–n heterojunction between ZnO and CuO has a high photocatalytic activity because of a better charge separation [16–22]. Liu et al. [23] prepared CuO/ZnO nanocomposites by homogeneous coprecipitation and used them for the photocatalytic degradation of methyl orange. Wei et al. [24] fabricated CuO/ZnO composite nanofilms using cathodic co-electrodeposition and observed their photocatalytic performance. Fierro et al. [25] synthesized CuO–ZnO composite catalysts by temperature-programmed reduction and applied them in photocatalytic degradation.

Nanocomposites loaded with metal oxide semiconductors have excellent optical, electrical, mechanical and chemical properties, which might result in applications in photocatalysis [26]. Electrospinning is a simple and convenient method for preparing composite nanofibers (CNFs) [27–31]. CNFs have been widely applied as carrier material due to their outstanding characteristics, such as high surface area, good thermal stability, and excellent mechanical properties [32–33]. CNFs loaded with metal oxide nanoparticles have attracted a great deal of attention regarding the photocatalytic purification of water. He et al. [34] fabricated porous graphene/TiO_2_ CNFs by electrospinning and observed their photocatalytic performance. Yuan et al. [35] obtained TiO_2_/WO_3_ CNFs using electrospinning and applied them in the photocatalytic removal of mercury. Teng et al. [36] prepared TiO_2_/NiO CNFs by electrospinning and used them for photocatalysis.

Polyacrylonitrile (PAN) has been widely used to fabricate nanofiber membranes because of its good spinnability, electrical conductivity, and heat resistance. However, carbonized PAN nanofiber membranes usually have poor mechanical properties. Polyvinylidene fluoride (PVDF) has better mechanical properties but a lower melting point. Carbonized PVDF/PAN CNFs have excellent mechanical properties due to the partial melting of PVDF after carbonization leading to point bonding. Therefore, blends of these two polymers were used as precursor for preparing the heterostructured CuO–ZnO-loaded CNF membranes (CNFMs) in our studies.

In our previous work [37], bubble-electrospinning was used to prepare Cu(Ac)_2_/Zn(Ac)_2_/PVDF/PAN on a large scale. Subsequently, PVDF/PAN CNFMs loaded with CuO and ZnO nanocrystals were prepared using heat treatment and hydrothermal synthesis. However, the diameter distribution of CNFMs obtained by bubble-electrospinning was not uniform because bubbles formed and the solution viscosity increased in the spinning process in an open environment. This resulted in poor mechanical properties of the fabricated PVDF/PAN CNFMs. In addition, the effects of the different components on morphology, structure, and properties of the CNFMs were not investigated, which is very important to obtain high-quality CNFMs.

Therefore, in the present study, heterostructured CuO–ZnO-loaded CNFMs were prepared using a combination of electrospinning, heat treatment, and hydrothermal synthesis. The effects of electrospinning, heat treatment, and hydrothermal synthesis parameters on morphology, structure, and properties of the CNFMs were investigated, and the optimal process parameters were determined. CuO–ZnO heterojunctions were successfully grown on the surface of PVDF/PAN CNFMs and were applied in the photocatalytic degradation of methyl orange.

## Experimental

### Materials

Anhydrous copper acetate (Cu(Ac)_2_, *M*_w_ = 199.65 g/mol) and anhydrous zinc acetate (Zn(Ac)_2_, *M*_w_ = 183.48 g/mol) were supplied by Shanghai Macklin Biochemical Co., Ltd. and Aladdin industrial Corporation (Shanghai, China), respectively. Polyacrylonitrile (PAN, *M*_w_ = 150,000 g/mol) powders were purchased from Beijing Lark Branch Co., Ltd. (Beijing, China). Polyvinylidene fluoride (PVDF, *M*_w_ = 400,000 g/mol) and *N*,*N*-dimethylformamide (DMF) were provided from Shanghai Chemical Reagent Co., Ltd. (Shanghai, China). Anhydrous copper sulfate (CuSO_4_, *M*_w_ = 159.60 g/mol), zinc chloride (ZnCl_2_, *M*_w_ = 73.09 g/mol), hexamethylenetetramine (C_6_H_12_N_4_, *M*_w_ = 140.19 g/mol) and ammonia (NH_3_·H_2_O, *M*_w_ = 17.03 g/mol) were supplied from China Pharmaceutical Group Chemical Reagents Co., Ltd. (Shanghai, China). Methyl orange (*M*_w_ = 327.34 g/mol) was purchased from Shanghai Debai Biotechnology Co., Ltd. (Shanghai, China). All materials were of analytical grade and applied without any further purification.

#### Preparation of heterostructured CuO–ZnO-loaded CNFMs

**Figure 1:**
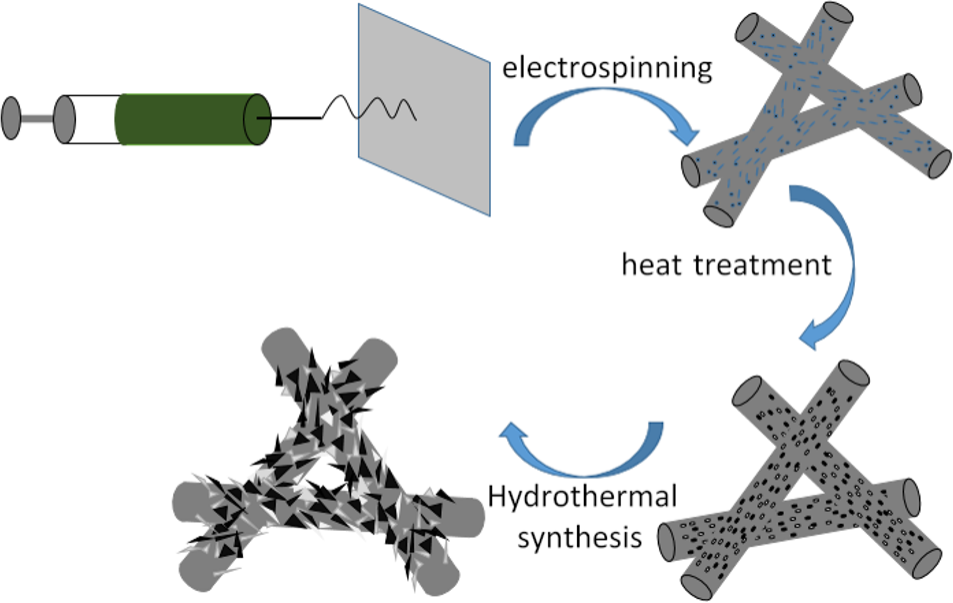
Schematic of the preparation of heterostructured CuO–ZnO-loaded CNFMs.

The heterostructured CuO–ZnO-loaded CNFMs were prepared by a combination of electrospinning, heat treatment and hydrothermal synthesis, as shown in Figure 1.

#### Electrospinning process

**Preparation of the spinning solution:** The mixed solutions of PVDF and PAN were prepared with 8 wt % by dissolving a mixture of PVDF and PAN with different weight ratios (9:1, 7:3, 5:5, 3:7 and 1:9) in DMF. The obtained solutions were magnetically stirred for 2 h at 80 °C until they became homogeneous. Then, equal amounts of Cu(Ac)_2_ and Zn(Ac)_2_ were added into the mixed solutions of PVDF and PAN, and were dispersed in the solutions by an ultrasonic cleaner (SL-5200DT, Nanjing Shunliu Instrument Co. Ltd., China) for 2 h at room temperature (25 ± 2 °C). The weight ratios of the added amounts of Cu(Ac)_2_ and Zn(Ac)_2_ were 1:5, 1:3 and 1:2.

**Preparation of electrospun Cu(Ac)_2_/Zn(Ac)_2_/PVDF/PAN CNFMs:** The prepared spinning solution was taken in a 10 mL syringe and delivered with a flow rate of 0.6 mL/h by a syringe pump. The applied voltage and the distance from needle tip to collector were, respectively, maintained at 20 kV and 18 cm. The electrospinning experiments were carried out at room temperature (25 ± 2 °C) and a relative humidity of (55 ± 5%), and the electrospun Cu(Ac)_2_/Zn(Ac)_2_/PVDF/PAN CNFMs were collected and carefully peeled off from the aluminium foil.

#### Heat-treatment process

The collected Cu(Ac)_2_/Zn(Ac)_2_/PVDF/PAN CNFMs were cut into pieces with a square area of 3 cm × 3 cm, and put into a muffle furnace (GZ2.5-10TP, Shanghai Gaozhi Precision Instrument Co., Ltd., China) for calcination. The heating rate was kept at 5 °C/min, and the temperatures varied from 80 to 180 °C.

A simple chemical solution strategy was used to form Cu(OH)_2_ and Zn(OH)_2_, and then CuO and ZnO nanoparticles were successfully obtained by the heat treatment process. During heat treatment of the electrospun Cu(Ac)_2_/Zn(Ac)_2_/PVDF/PAN nanofiber membranes, Cu(Ac)_2_ and Zn(Ac)_2_ in the membrane were in contact with water vapor and directly decomposed to form CuO, ZnO and evaporated acetic acid. All reactions were carried out under heating conditions, such as heat treatment and hydrothermal synthesis. The whole process can be described by the following equations [16].


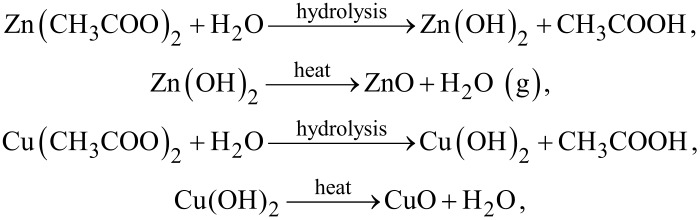


or


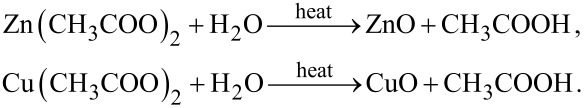


#### Hydrothermal process

**Preparation of growth solution:** The growth solutions used in the hydrothermal process were obtained by preparing mixtures of C_6_H_12_N_4_, NH_3_·H_2_O, and a saturated aqueous solution of CuSO_4_ and ZnCl_2_. The saturated solution of CuSO_4_ and ZnCl_2_ was prepared by dissolving an excess of CuSO_4_ and ZnCl_2_ in 100 mL deionized water. Next, the saturated solution was diluted according to the following scheme. To one part of the saturated solution 1, 4, or 24 parts of deionized water were added to obtain dilutions of 1:2, 1:5, or 1:25, respectively. To aliquots of the saturated solution and of the three diluted solutions, C_6_H_12_N_4_ powder was added in excess under stirring. Subsequently, NH_3_·H_2_O was dripped into the above solutions and stirred fully until the solutions became clear. Finally, 3–5 drops of NH_3_·H_2_O were added to the four solutions, and the four prepared growth solutions were sealed for use in the hydrothermal process.

**Preparation of heterostructured CuO–ZnO-loaded CNFMs:** The calcined CNFMs were fixed on glass sheets and put in four 50 mL reaction kettles that contained 10 mL of the above four growth solutions. After tightening the cover, the reaction kettles were placed in an electrothermal oven (DHG-9030A, Shanghai Shenxian Constant Temperature Equipment Factory, China). At the end of the set reaction time, the fabricated heterostructured CuO–ZnO-loaded CNFMs were taken out with tweezers, and were rinsed repeatedly with deionized water for several times. Next, the rinsed CNFMs were dried in the electrothermal oven, and then the dried CNFMs were packed in self-sealing bags for subsequent tests and applications.

The growth mechanism of CuO and ZnO nanocrystals on the calcined CNFMs using hydrothermal synthesis is as follows. When C_6_H_12_N_4_ is added to the solution, a large amount of ammonium ions will be released, and copper ions and zinc ions will form [Cu(NH_3_)_4_]^2+^ and [Zn(NH_3_)_4_]^2+^, respectively. Finally, Cu(OH)_2_ and Zn(OH)_2_ are formed under alkaline conditions after the addition of NH_3_·H_2_O. At a certain temperature and pressure, the water evaporate, and CuO and ZnO nanocrystals will grow on the calcined CNFMs. The growth mechanism can also be expressed by the following chemical equations.


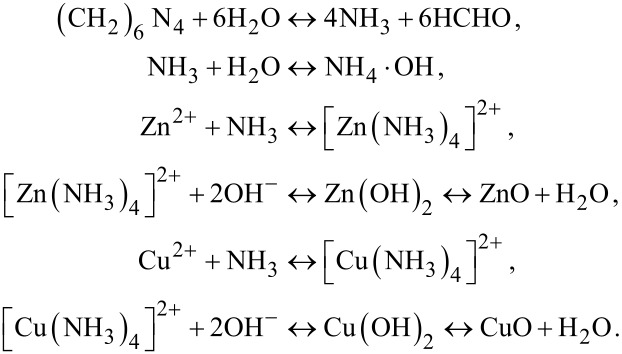


As reference, CuO-loaded PVDF/PAN CNFMs and ZnO-loaded PVDF/PAN CNFMs were also prepared using the same method.

#### Measurement and characterization

Diameter and arrangement of CNFMs were investigated using scanning electron microscopy (SEM, Hitachi S-4800, Japan). The matrix morphology and fiber diameter distribution were determined using the Image J software (National Institute of Mental Health, Bethesda, Maryland, USA). The diameter of 100 randomly selected fibers in each sample were chosen for diameter characterization, including the measured diameter distribution histogram, as well as the calculated average diameter, standard deviation, and confidence interval. In addition, energy dispersion spectroscopy (EDS, Hitachi S-4800, Japan) was used to identify the elemental composition of the sample regions evaluated with SEM. The pore size distributions of CNFMs were measured using capillary flow porometry (Porometer 3G, Quantachrome Instruments, USA). All samples were circular membranes with a diameter of 25 mm and the thickness of 10 μm. FTIR spectra of CNFMs were obtained using Fourier-transform infrared spectroscopy (FTIR, Nicolet5700, Thermo Nicolet Company, Waltham, MA, USA), carrying out 32 scans within the wavenumber range of 400–4000 cm^−1^ with a resolution of 4 cm^−1^. X-ray diffraction (XRD) analyses were carried out using a Philips X’Pert-Pro MPD (PANalytical, Almelo & Eindhoven, Netherlands). The mechanical properties of CNFMs were characterized using a universal electromechanical test machine Instron-3365 (Instron, Norwood, MA, USA). All samples were 4 cm × 1 cm rectangular membranes, and the measurement of each sample was repeated five times. Contact angle (CA) measurements of CNFMs were carried out using a Krüss K100 apparatus (Krüss Company, Hamburg, Germany). A droplet of 6 μL deionized water was used for static CA measurements, and the average CAs were determined by measuring five different positions of the same sample.

#### Photocatalytic degradation

Photocatalytic degradation processes under ultraviolet (UV) irradiation were investigated using the prepared CNFMs degrading a methyl orange solution with an initial concentration of 10 mg/L. The prepared CNFMs (0.05 g) were added to 30 mL of the methyl orange solution, and then the solution was placed in a dark environment for 2 h to achieve an adsorption–desorption equilibrium. Next, photocatalytic degradation of methyl orange was carried out under UV irradiation (λ = 254 nm). The UV lamp had a light intensity of 1.1 mW/cm, which was measured by a radiometer. The degradation rate of methyl orange was determined by measuring the maximum absorbance of methyl orange at 465 nm using a UV spectrophotometer (Cary 5000, Agilent Technologies, USA). Each photocatalytic degradation experiment was performed three times.

## Results and Discussion

### Characterization of electrospun Cu(Ac)_2_/Zn(Ac)_2_/PVDF/PAN CNFMs

Cu(Ac)_2_/Zn(Ac)_2_/PVDF/PAN CNFMs were fabricated using electrospinning, and the effects of different component contents on morphology, structure and properties of the CNFMs were investigated.

#### Effect of the weight ratio PVDF/PAN on the electrospun CNFMs

Electrospun Cu(Ac)_2_/Zn(Ac)_2_/PVDF/PAN CNFMs with a weight ratio [Cu(Ac)_2_/Zn(Ac)_2_]/[PVDF/PAN] = 1:3 were prepared, and the effects of the weight ratio of PVDF to PAN on the CNFMs were examined.

**Figure 2:**
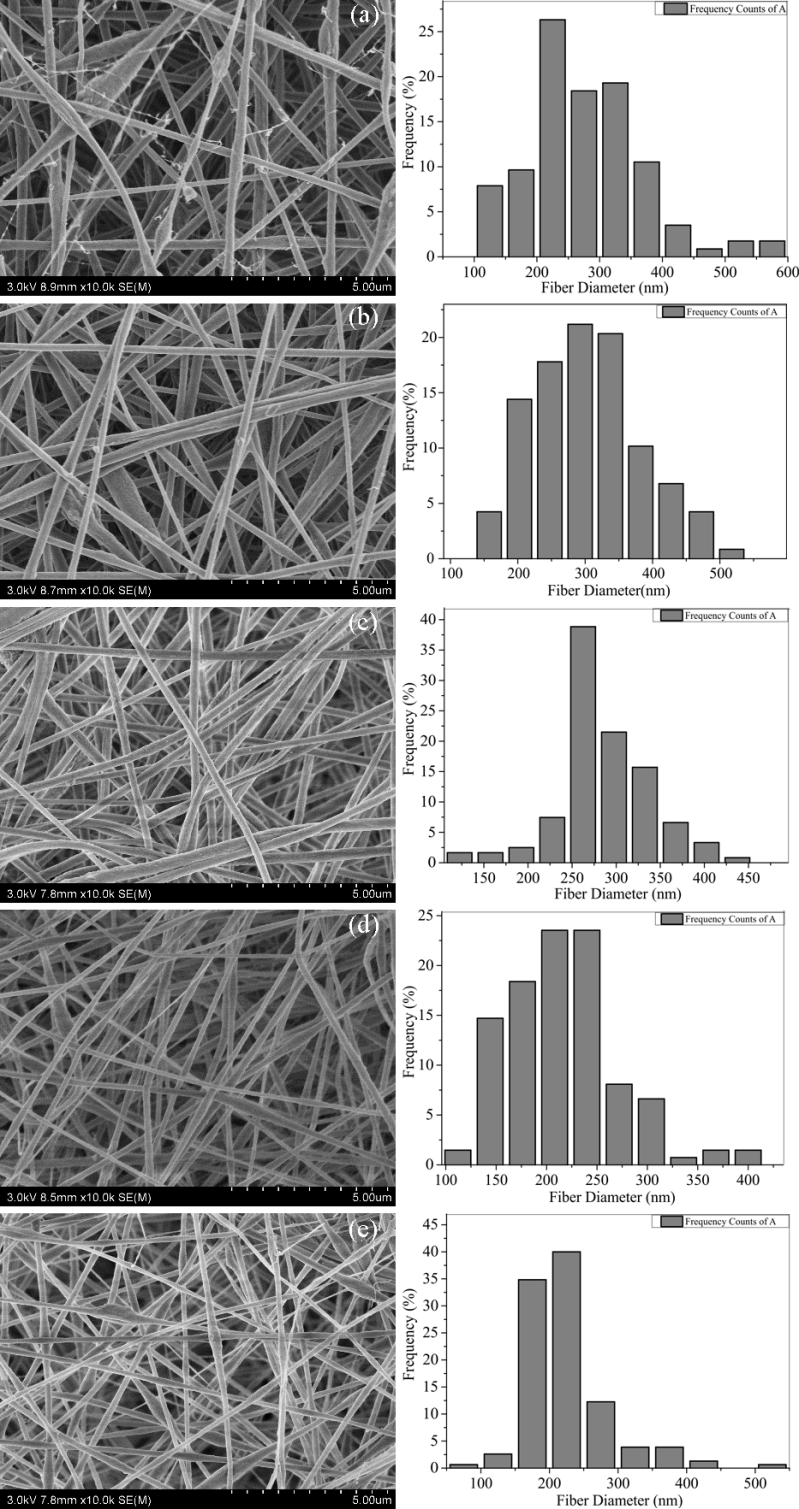
SEM images of electrospun CNFMs with different PVDF/PAN weight ratios: (a) 9:1; (b) 7:3; (c) 5:5; (d) 3:7; (e) 1:9. On the right-hand side are the corresponding nanofiber diameter distributions.

**Morphological characterization:** The morphology of the CNFMs with different weight ratios of PVDF to PAN was characterized by SEM. The SEM images of the electrospun CNFMs and the according nanofiber diameter distributions are presented in Figure 2. The nanofiber diameters of the CNFMs as a function of the PVDF/PAN weight ratio are given in Table 1. The average nanofiber diameters of the CNFMs are all in the range of 200–300 nm, and with a decrease of the weight ratio, the average nanofiber diameters of the CNFMs decrease because of the decreased average molecular weight [22]. At a weight ratio of 1:9, the average nanofiber diameter increases slightly because of the appearance of beads (Figure 2e).

**Table 1:** Average nanofiber diameters of the electrospun CNFMs with different PVDF/PAN weight ratios.

PVDF/PAN	average diameter (nm)	standard deviation (nm)	confidence interval (nm)

9:1	275	94.0	±18.4
7:3	302	80.8	±15.8
5:5	284	51.5	±10.1
3:7	216	82.0	±16.1
1:9	227	60.1	±12.0

Figure 2 shows that at a PVDF/PAN weight ratio of 9:1, the nanofiber diameter distribution is non-uniform, and filaments and beads are observable. As the weight ratio decreases, the number of filaments and beads decreases significantly. At a ratio of 5:5, the nanofiber diameter distribution of the CNFMs is uniform, and there are no filaments and beads. With further decreasing weight ratio, filaments and beads appear again, resulting in a non-uniform nanofiber diameter distribution. Therefore, a weight ratio of 5:5 was selected as the optimal parameter for the following experiments.

**Figure 3:**
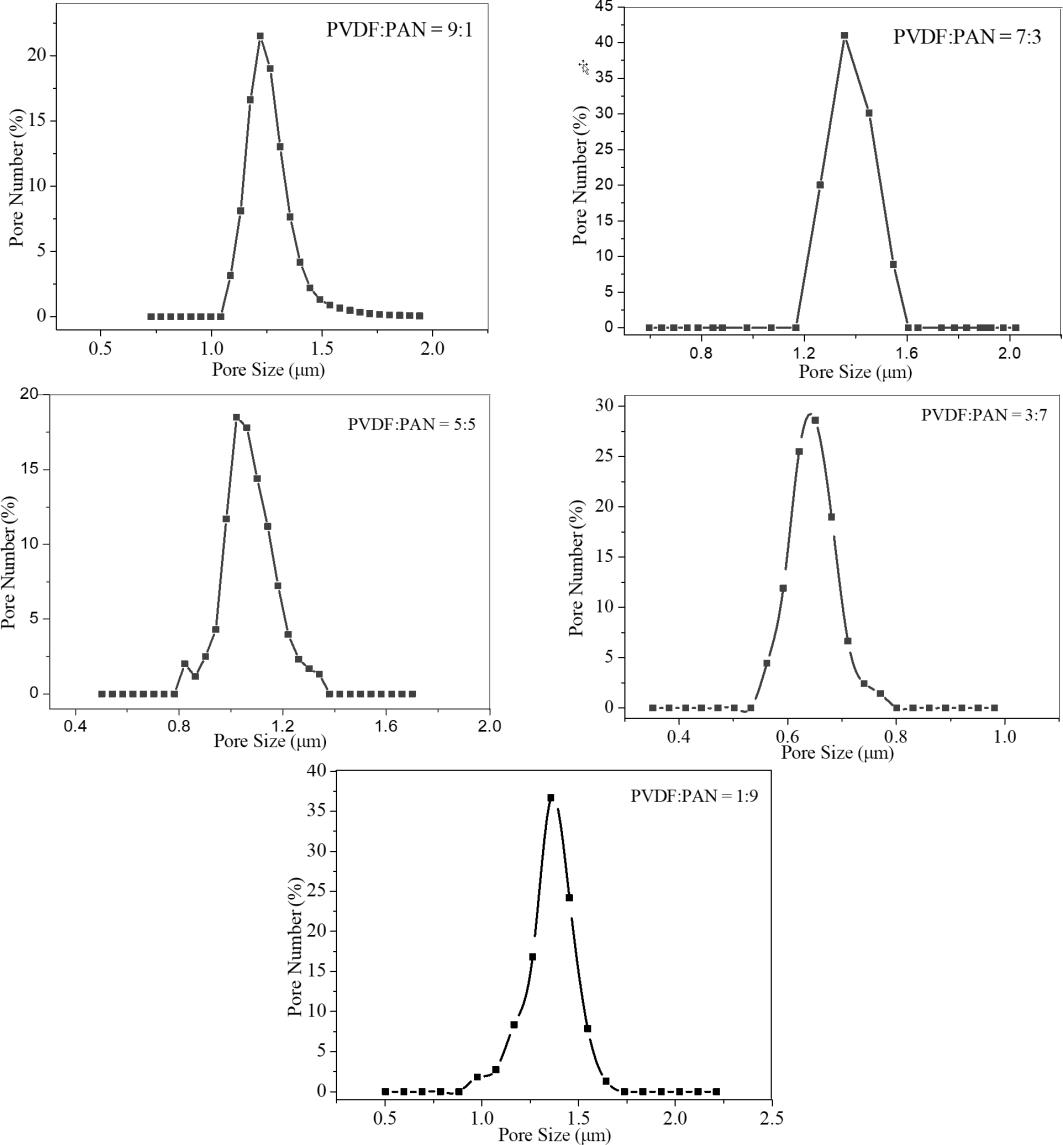
Pore size distributions of CNFMs with different PVDF/PAN weight ratios.

**Pore size distribution of the electrospun CNFMs:** The pore size distribution of the CNFMs with different PVDF/PAN weight ratios determined by capillary flow porometry are illustrated in Figure 3, and the calculated data are given in Table 2. At a weight ratio of 3:7 the pore sizes of the CNFMs were the smallest, and at a weight ratio of 7:3 the pore sizes of the CNFMs were the largest. Taking into account the nanofiber diameters of the CNFMs presented in Table 1, it can be found that the pore sizes of the CNFMs are mainly determined by the nanofiber diameters. Larger nanofiber diameters lead to larger pore sizes.

**Table 2:** Calculated pore size distributions of the CNFMs with different PVDF/PAN weight ratios.

PVDF/PAN	pore size (μm)	pore size corresponding to the highest number of pores (μm)

9:1	1.087–1.781	1.363
7:3	1.262–1.642	1.452
5:5	0.822–1.342	1.062
3:7	0.562–0.771	0.681
1:9	0.977–1.642	1.357

**Figure 4:**
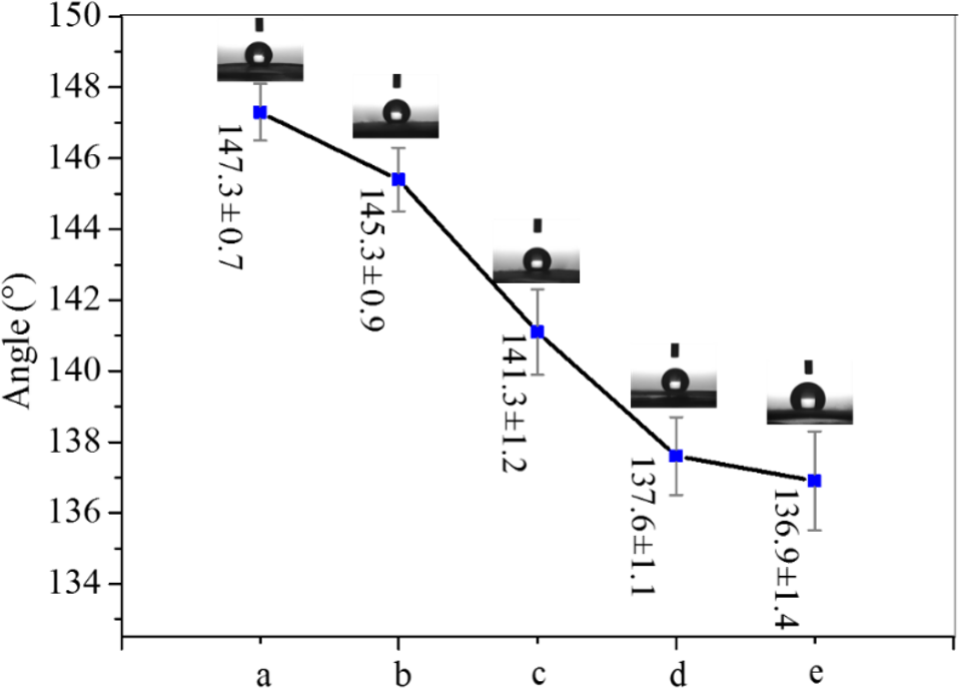
Contact angles of CNFMs with different PVDF/PAN weight ratios: (a) 9:1; (b) 7:3; (c) 5:5; (d) 3:7; (e) 1:9.

**Wetting properties:** The measured contact angle (CA) values of the CNFMs with different PVDF/PAN weight ratios are displayed in Figure 4. All CNFMs are hydrophobic, and the hydrophobicity of the CNFMs decreases slightly with decreasing weight ratio. The reason might be that the hydrophobicity of pure PVDF nanofiber membranes (NFMs) is higher than that of pure PAN NFMs. Also, nanofiber diameter distribution, thickness and surface roughness of the CNFMs can lead to the difference in hydrophobicity.

**Figure 5:**
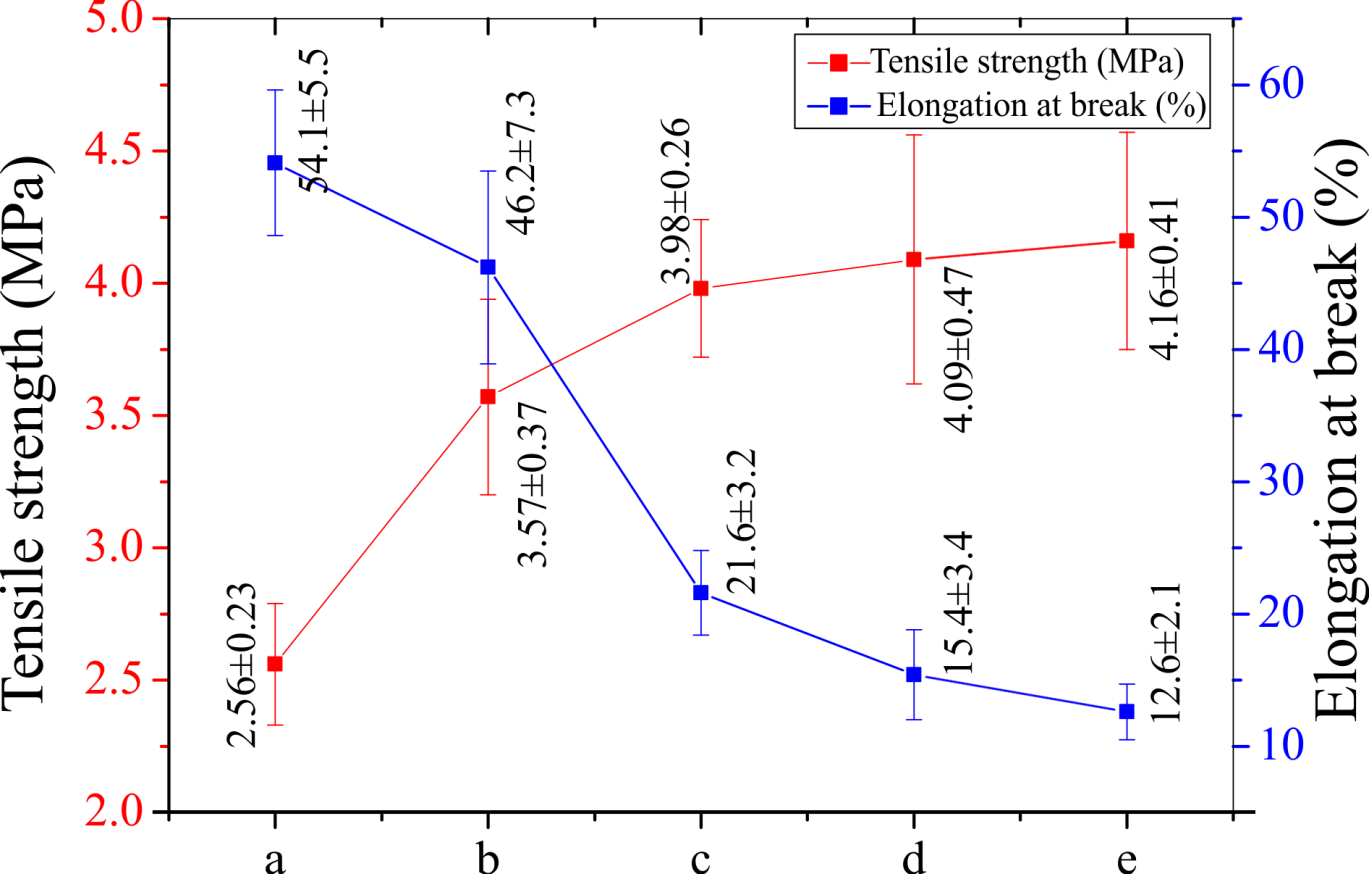
Mechanical properties of the CNFMs with different PVDF/PAN weight ratios: (a) 9:1; (b) 7:3; (c) 5:5; (d) 3:7; (e) 1:9.

**Mechanical properties:** Mechanical properties of the CNFMs with different PVDF/PAN weight ratios are exhibited in Figure 5. With the decrease of the weight ratio the breaking elongation of the CNFMs decreases gradually and the tensile strength increases slowly, resulting in better strength and poorer flexibility of the CNFMs. Therefore, CNFMs with relatively good mechanical properties could be obtained by blending PVDF and PAN at a certain weight ratio.

#### Effect of the additive content of Cu(Ac)_2_ and Zn(Ac)_2_ on the CNFMs

Electrospun Cu(Ac)_2_/Zn(Ac)_2_/PVDF/PAN CNFMs with a weight ratio of PVDF/PAN = 5:5 were fabricated, and the effects of the weight ratio of added Cu(Ac)_2_ and Zn(Ac)_2_ were examined.

**Figure 6:**
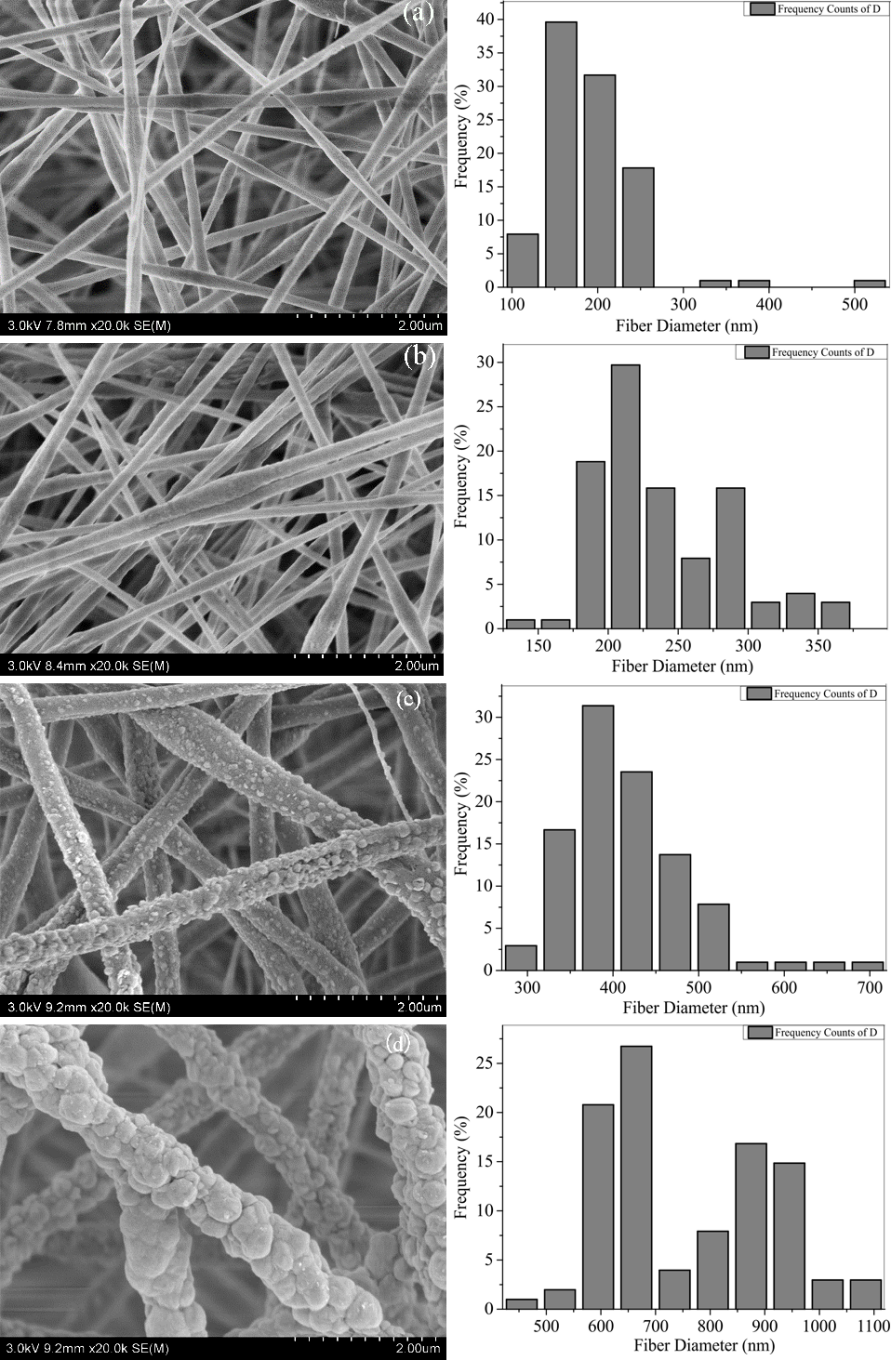
SEM images of the electrospun CNFMs with different weight ratios of Cu(Ac)_2_/Zn(Ac)_2_ to PAN/PVDF: (a)1:5; (b) 1:3; (c) 1:2; (d) 1:1. On the right-hand side are the corresponding nanofiber diameter distributions.

**Morphological characterization:** SEM images of the CNFMs with different weight ratios between Cu(Ac)_2_/Zn(Ac)_2_ and PAN/PVDF are presented in Figure 6, including the corresponding nanofiber diameter distributions. The nanofiber diameters of the CNFMs as a function of the weight ratio [Cu(Ac)_2_/Zn(Ac)_2_]/[PVDF/PAN] are given in Table 3.

**Table 3:** Average nanofiber diameters of electrospun CNFMs with different [Cu(Ac)_2_/Zn(Ac)_2_]/[PVDF/PAN] weight ratios.

[Cu(Ac)_2_/Zn(Ac)_2_]/[PVDF/PAN]	average diameter (nm)	standard deviation (nm)	confidence interval (nm)

1:5	193	52.7	±10.3
1:3	236	47.9	±9.4
1:2	414	70.8	±13.9
1:1	758	148.5	±29.1

Figure 6 and Table 3 show that, as the [Cu(Ac)_2_/Zn(Ac)_2_]/[PVDF/PAN] weight ratio increases, the average nanofiber diameters of the CNFMs increase and the surface of the nanofibers changes from smooth to rough. This is because when only small amounts of Cu(Ac)_2_ and Zn(Ac)_2_ are added, only very small particles are formed. But with increasing amounts of added Cu(Ac)_2_ and Zn(Ac)_2_ larger particles are gradually appearing on the surface of the nanofibers. When the weight ratio [Cu(Ac)_2_/Zn(Ac)_2_]/[PVDF/PAN] was 1:5 or 1:3, only few and small Cu(Ac)_2_ and Zn(Ac)_2_ particles are formed on the surface of the nanofibers. At a weight ratio of 1:2, larger particles are evenly distributed on the surface of the nanofibers, which provide a seed layer for further hydrothermal growth. When the weight ratio reaches 1:1, the nanofibers the particles aggregated and the fibers become bundled. Therefore, a weight ratio of 1:2 was chosen as the optimum parameter for further studies.

**Figure 7:**
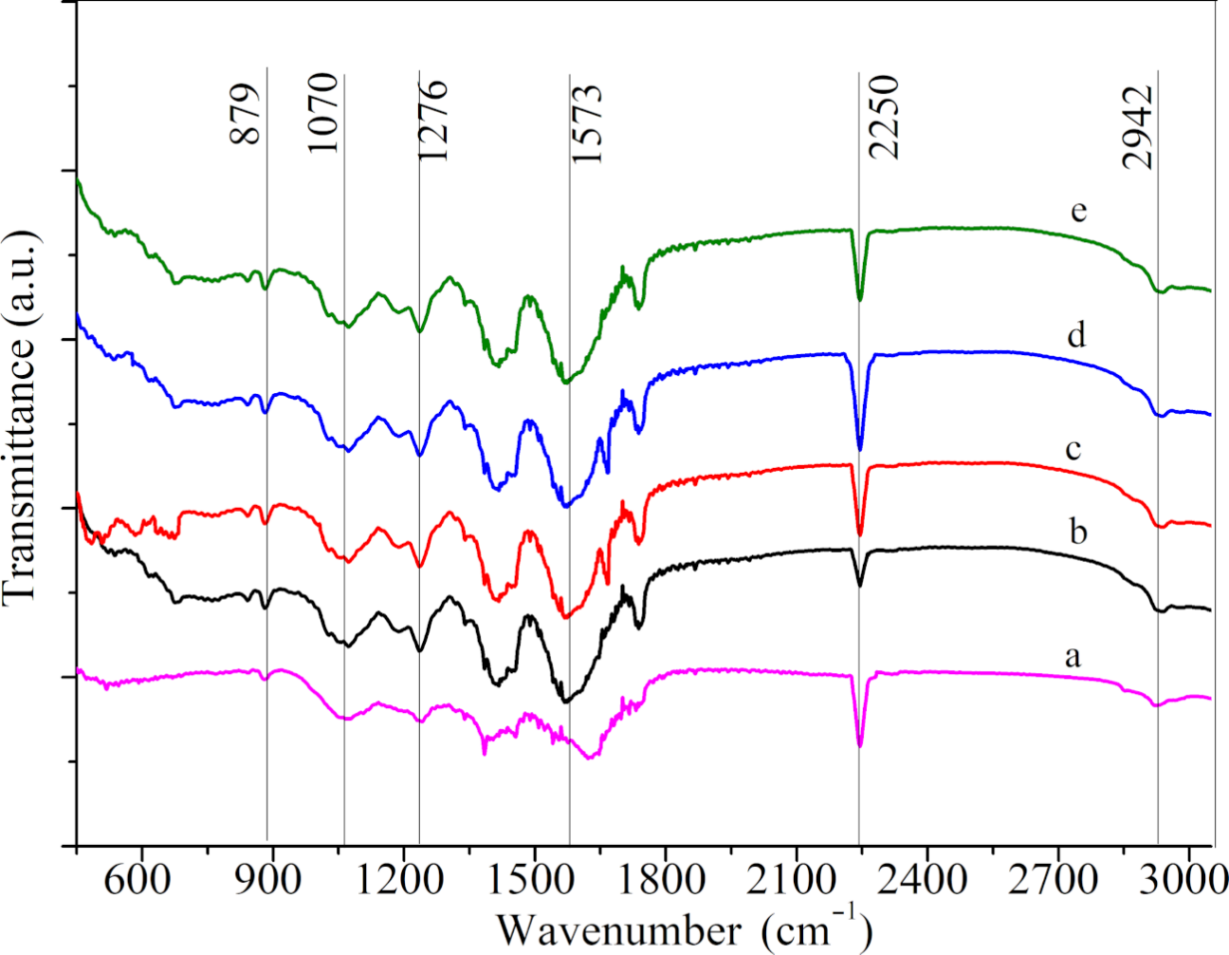
FTIR spectra of the CNFMs with different weight ratios [Cu(Ac)_2_/Zn(Ac)_2_]/[PVDF/PAN]: (a) 0:1; (b) 1:5; (c) 1:3; (d) 1:2; (e) 1:1.

**FTIR and XRD analysis:** FTIR was used to characterize the CNFMs with different [Cu(Ac)_2_/Zn(Ac)_2_]/[PVDF/PAN] weight ratios (Figure 7). The peaks at 879 cm^−1^ correspond to the asymmetric stretching vibration of –CF_2_– in PVDF. The peaks at 1070 and 1276 cm^−1^ represent the β-phase of PVDF. There was also an obscure peak near 1070 cm^−1^, which might belong to –C–C– stretching vibrations. The peak at 2250 cm^−1^ corresponds to the stretching vibration of –CN– in PAN and the peak at 2942 cm^−1^ can be assigned to the stretching vibration of –CH_2_–. The spectra of the CNFMs with Cu(Ac)_2_ and Zn(Ac)_2_ (Figure 7b–e) have a wide peak at 1573 cm^−1^, which represents the antisymmetric stretching vibration of –COO–, indicating that these CNFMs include Cu(Ac)_2_ and Zn(Ac)_2_ [38–40]. The change of the [Cu(Ac)_2_/Zn(Ac)_2_]/[PVDF/PAN] weight ratio has little influence on the spectra.

**Figure 8:**
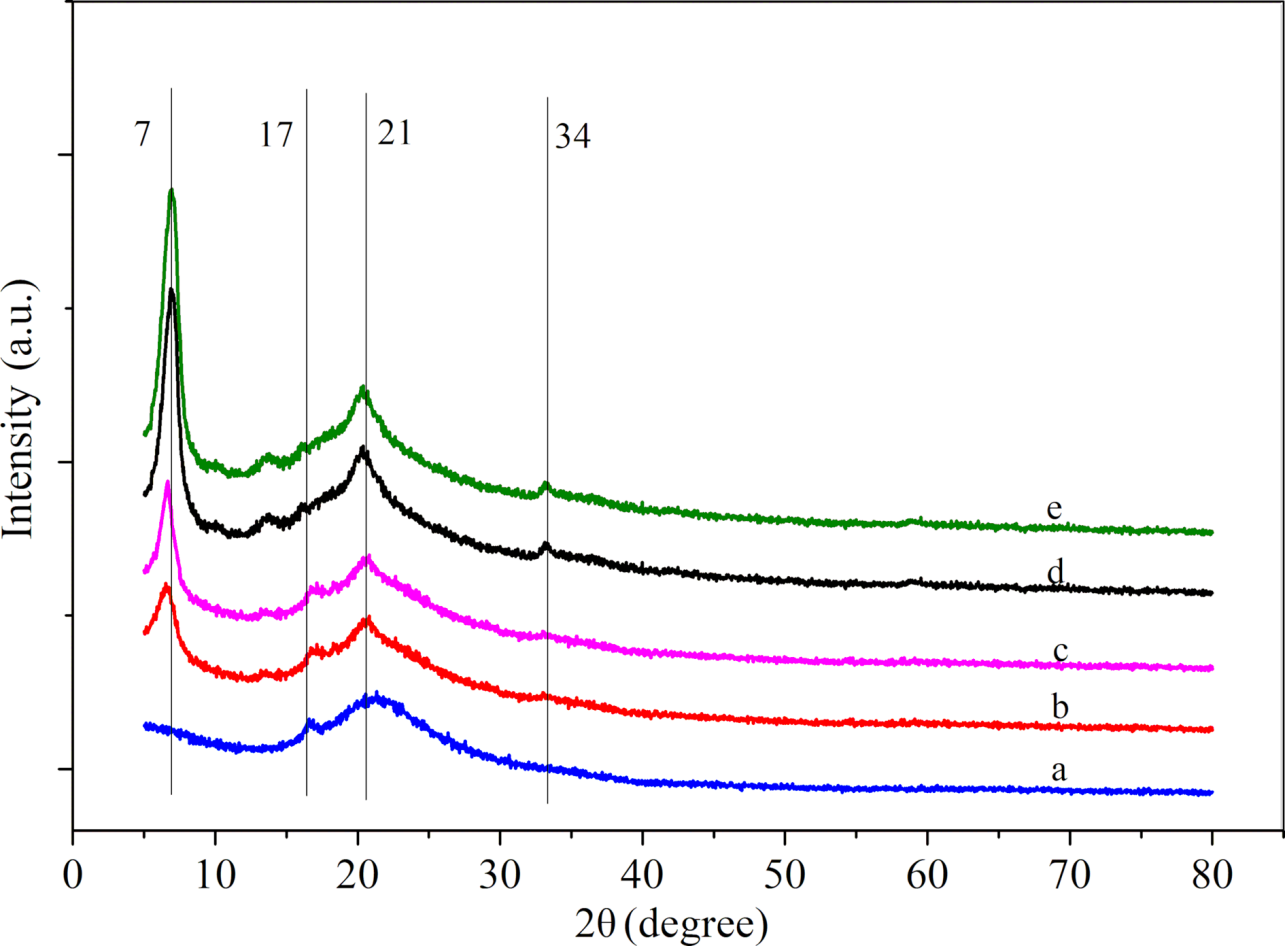
XRD spectra of the CNFMs with different [Cu(Ac)_2_/Zn(Ac)_2_]/[PVDF/PAN] weight ratios: (a) 0:1; (b) 1:5; (c) 1:3; (d) 1:2; (e) 1:1.

**Figure 9:**
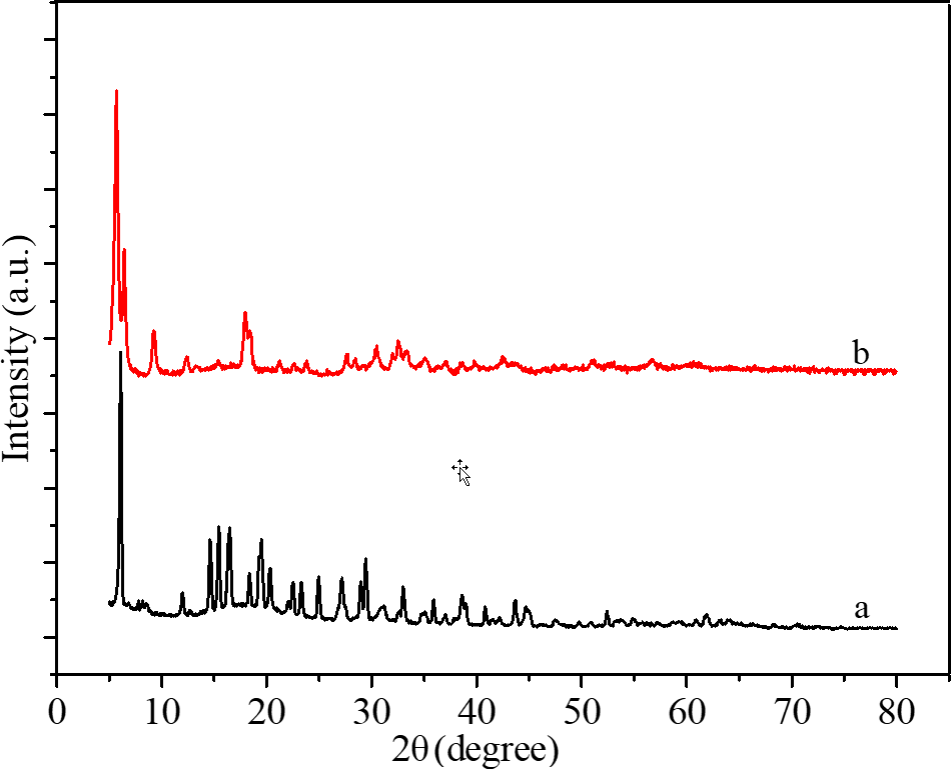
XRD spectra of (a) pure Cu(Ac)_2_ powder and (b) pure Zn(Ac)_2_ powder.

In order to determine the influence of the [Cu(Ac)_2_/Zn(Ac)_2_]/[PVDF/PAN] weight ratio on the crystallinity of the CNFMs, XRD analyses were performed and the XRD patterns are shown in Figure 8 and Figure 9. Pure PVDF/PAN CNFMs show the characteristic diffraction peaks at 17° and 21° (Figure 8a). The pure Cu(Ac)_2_ and Zn(Ac)_2_ powders exhibit diffraction peaks at 7° (Figure 9). Also, the CNFMs with Cu(Ac)_2_ and Zn(Ac)_2_ (Figure 8b–e) have obvious diffraction peaks at 7°. As the amount of Cu(Ac)_2_ and Zn(Ac)_2_ increases, the intensity of the peaks corresponding to Cu(Ac)_2_ and Zn(Ac)_2_ in the patterns of the CNFMs also increases. In addition, diffraction peaks at 34° appear and increase gradually. At the same time, the diffraction peaks at 21° gradually change from smooth to sharp, which might be due to the superposition of the diffraction peaks of PVDF with those of Cu(Ac)_2_ and Zn(Ac)_2_ between 5° and 20° [13,41–42]. The XRD results indicate that there are no new crystalline phases in the CNFMs, and that Cu(Ac)_2_, Zn(Ac)_2_, PVDF and PAN retain their crystalline structure.

#### Characterization of PVDF/PAN CNFMs with CuO and ZnO nanoparticles

The electrospun PVDF/PAN/Cu(Ac)_2_/Zn(Ac)_2_ CNFMs with the optimum weight ratios (Cu(Ac)_2_/Zn(Ac)_2_ = 1:1, PVDF/PAN = 5:5 and [Cu(Ac)_2_/Zn(Ac)_2_]/[PVDF/PAN] = 1:2) were calcined and PVDF/PAN CNFMs with CuO and ZnO nanoparticles were obtained. The effects of heat-treatment temperature and time on morphology, structure and properties of the CNFMs were characterized.

#### Effect of the heat-treatment temperature on the CNFMs

To illustrate the effects of heat-treatment temperature on the morphology of the CNFMs, the temperatures set to 80, 100, 120, 140, 160 and 180 °C. The heat-treatment time was 2 h in all cases.

**Figure 10:**
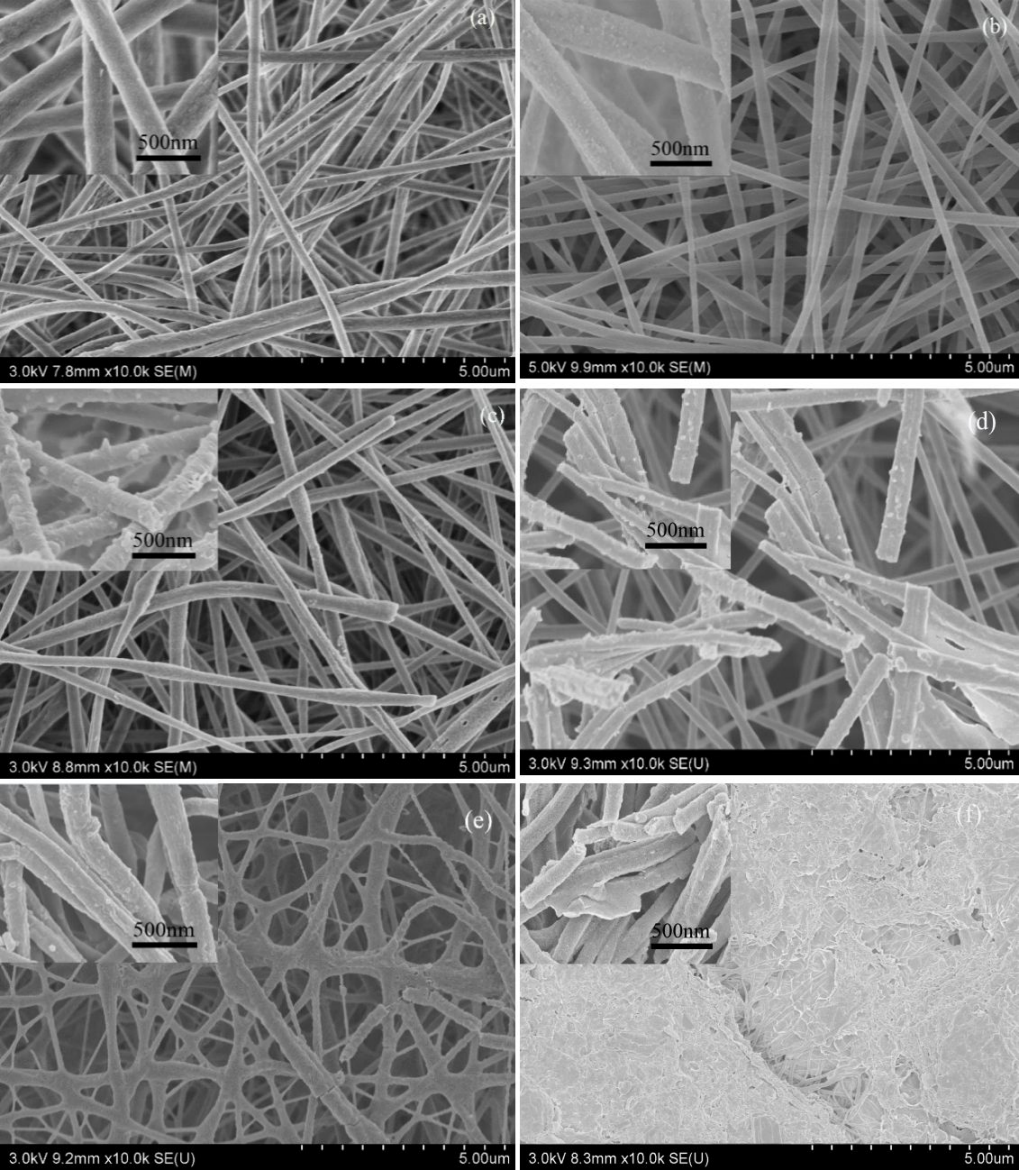
SEM images of PVDF/PAN CNFMs with CuO and ZnO nanoparticles obtained after 2 h of heat treatment at different temperatures: (a) 80 °C; (b) 100 °C; (c) 120 °C; (d) 140 °C; (e) 160 °C; (f) 180 °C.

**Morphological characterization:** Figure 10 shows SEM images of PVDF/PAN CNFMs with CuO and ZnO nanoparticles obtained with different heat-treatment temperatures after 2 h. For heat-treatment temperatures of 80 and 100 °C, the morphology of the CNFMs has changed only little compared to that of the CNFMs without heat treatment. The surface is still smooth and the nanofibers are intact. When the temperature is 120 °C, the nanofibers begin to break due to the partial melting of PVDF. When the temperature reaches 140 °C, the nanofibers begin to shrink and bend, but they remain fibrous. At a temperature of 160 °C, most of the nanofibers begin to melt. Finally, when the temperature reaches 180 °C, PVDF completely melts and the CNFMs lose the characteristics of nanofibers.

As the temperature increases, CuO and ZnO nanoparticles formed by thermal decomposition of Cu(Ac)_2_ and Zn(Ac)_2_ appear. Figure 10c,d show that after heat treatment at temperatures of 120–140 °C, there are many evenly distributed CuO and ZnO nanoparticles on the surface of the nanofibers. When the temperature reaches 160 °C, the number and size of nanoparticles on the surface decreases (Figure 10e). When the temperature is 180 °C, the nanoparticles disappear completely (Figure 10f), because the molten PVDF covers the nanoparticles. Hence, the temperature range from 120 to 140 °C yields CNFMs with CuO and ZnO seeds that can act good templates for the subsequent hydrothermal growth.

**Figure 11:**
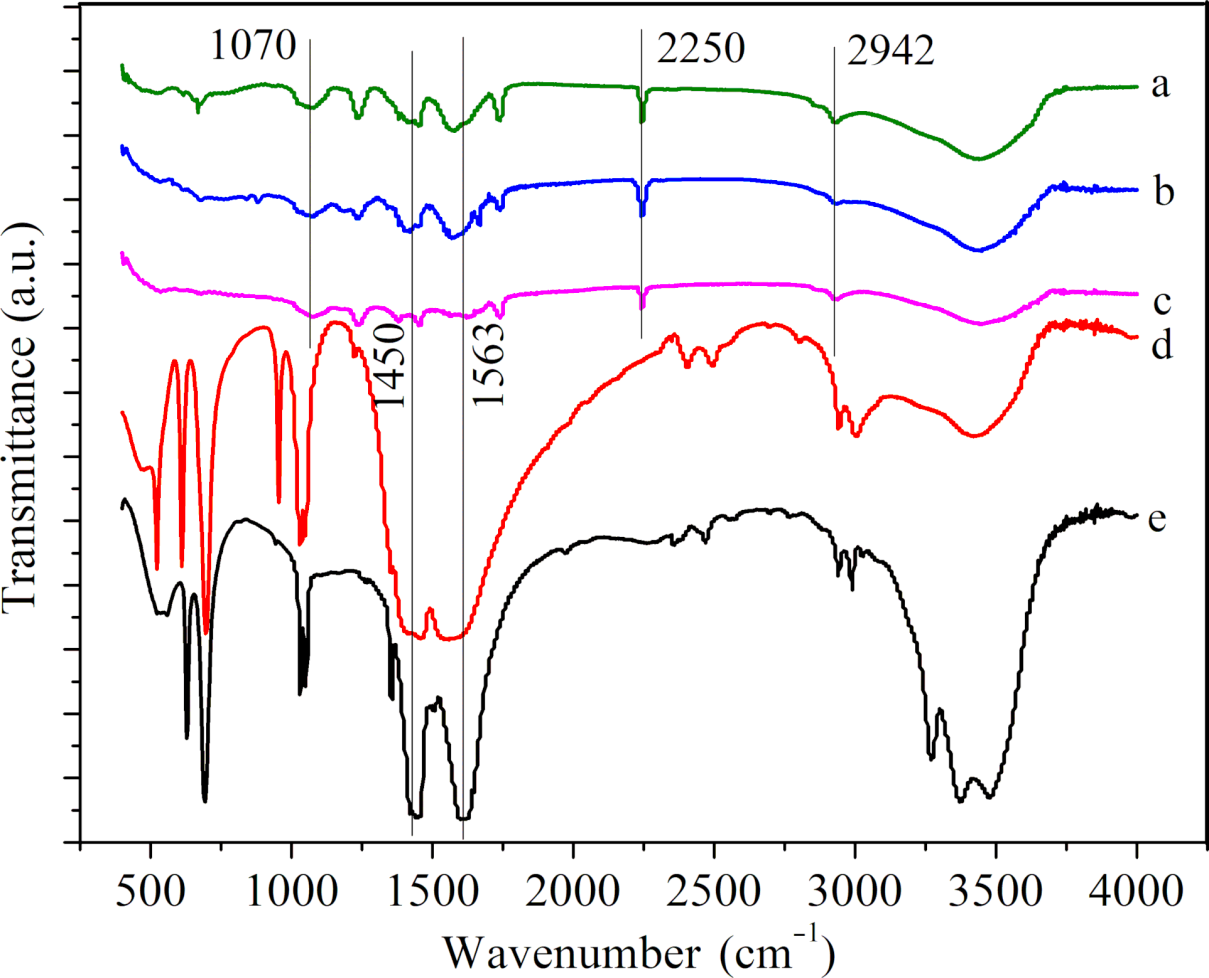
FTIR spectra of (a) CNFMs with heat treatment for 2 h at 140 °C; (b) CNFMs without heat treatment; (c) PVDF/PAN CNFMs; (d) Cu(Ac)_2_ powder; (e) Zn(Ac)_2_ powder.

**FTIR and XRD analysis:** CNFMs with and without heat treatment were analyzed by FTIR (Figure 11). The spectra of the CNFMs with and without heat treatment (Figure 11a, b) as well as of pure PVDF/PAN CNFMs (Figure 11c) show three peaks at 1070, 2250 and 2942 cm^−1^.These three peaks represent the stretching vibration of –C–C– in the β-phase of PVDF, the stretching vibration of –CN– in PAN and the telescopic vibration of –CH_2_– in PAN, respectively. By comparison, we conclude that the heat treatment at 140 °C has little effect on PVDF and PAN in the fibrous membranes, probably because the melting temperature was not reached. Also, the spectra of the CNFMs with and without heat treatment (Figure 11a,b) as well as those of Cu(Ac)_2_ and Zn(Ac)_2_ powders (Figure 11d,e) had peaks at 1563 and 1450 cm^−1^, which represent the symmetric and asymmetric vibrations of –COO–. However, the corresponding peaks of the CNFMs after heat treatment for 2 h at 140 °C (Figure 11a) are slightly weaker than with those of the CNFMs without heat treatment (Figure 11b), which indicates that Cu(Ac)_2_ and Zn(Ac)_2_ reacted during the heat treatment process, and it is likely that CuO and ZnO nanoparticles formed.

**Figure 12:**
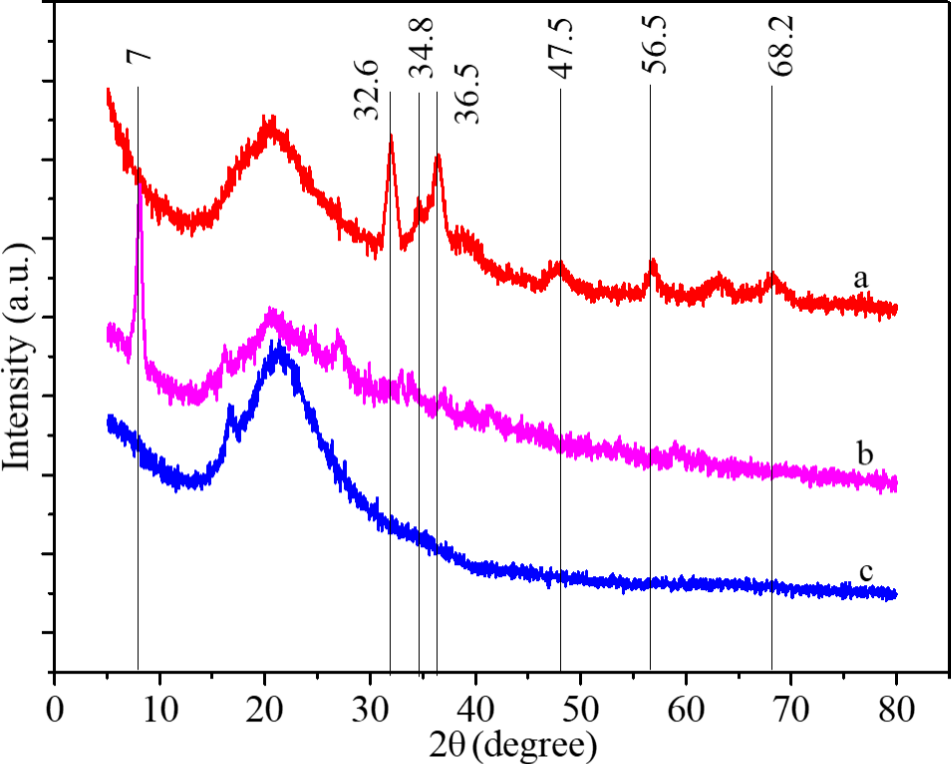
XRD spectra of (a) the CNFMs after heat treatment for 2 h at 140 °C; (b) the CNFMs without heat treatment; (c) PVDF/PAN CNFMs.

XRD analyses were performed to determine the effect of the heat treatment on the crystallinity of the CNFMs. The XRD patterns with distinctive crystalline peaks are given in Figure 12. It can be seen that the CNFMs without heat treatment (Figure 12b) have a diffraction peak at 7°. The CNFMs that were heat-treated for 2 h at 140 °C (Figure 12a) show diffraction peaks at 32.6°, 34.8°, 36.5°, 47.5°, 56.5° and 68.2°, corresponding to the characteristic peaks of CuO and ZnO [40]. This shows that Cu(Ac)_2_ and Zn(Ac)_2_ in the CNFMs had been transformed to CuO and ZnO, respectively, during the heat treatment process, and CuO and ZnO nanoparticles were formed.

**Figure 13:**
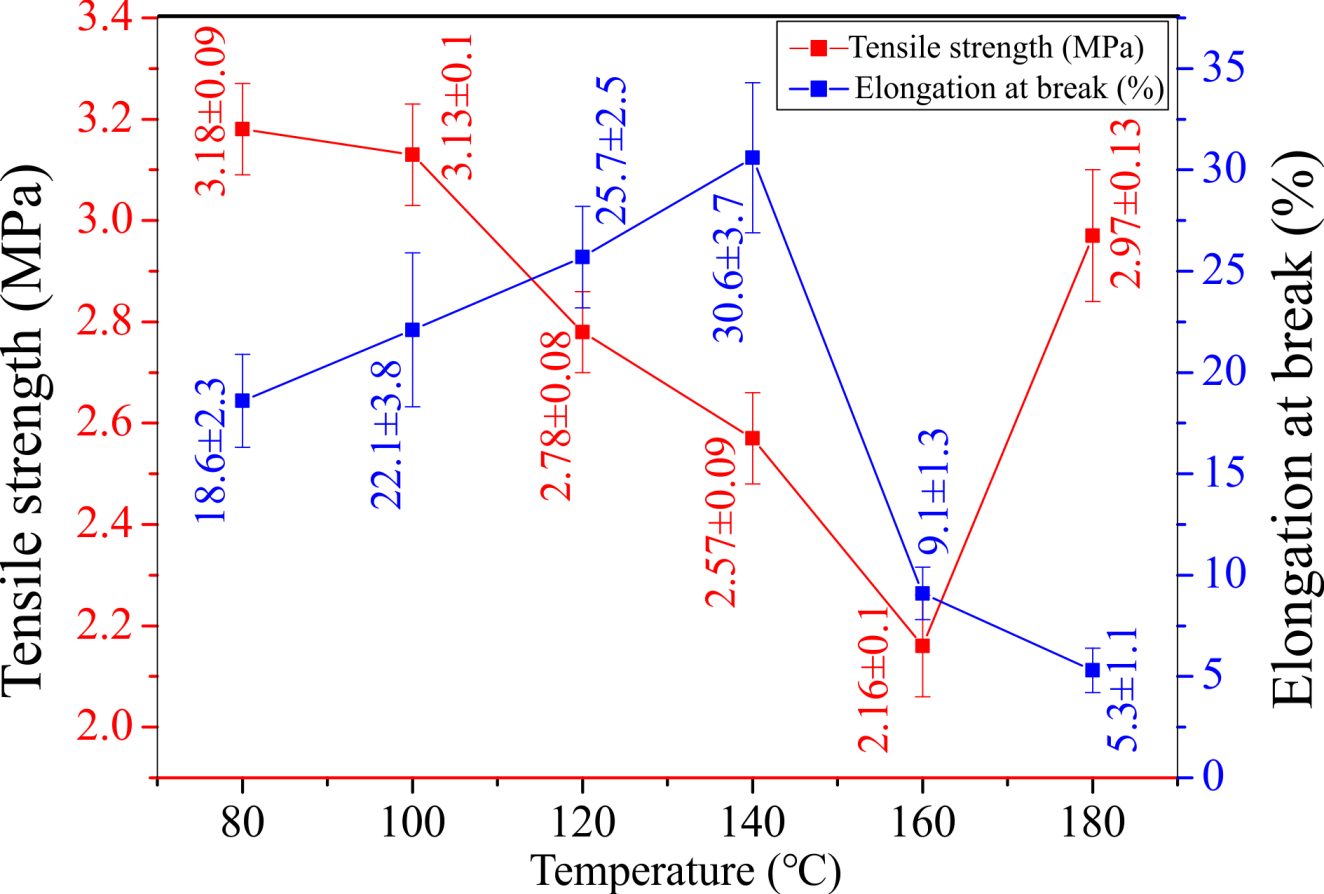
Mechanical properties of the CNFMs obtained after heat treatment for 2 h at different temperatures: (a) 80 °C; (b) 100 °C; (c) 120 °C; (d) 140 °C; (e) 160 °C; (f) 180 °C.

**Mechanical properties:** The mechanical properties of PVDF/PAN CNFMs with CuO and ZnO nanoparticles prepared with heat-treatment at different temperatures for 2 h are displayed in Figure 13. In the temperature range from 80 to 160 °C, the tensile strength of the CNFMs decreases gradually with increasing heat-treatment temperature, due to the change of the nanofiber structure caused by the produced CuO and ZnO nanoparticles and the gradual melting of PVDF. At the same time, the carbon chain of the PAN molecules vibrated violently and arranged more neatly after the heat treatment, resulting in a decrease of tensile strength and the fracture of brittle nanofibers under the action of external force. However, when the temperature reached 180 °C, the tensile strength of the CNFMs increased suddenly because of the increased adhesion between nanofibers resulting from the almost complete melting of PVDF.

In addition, as the heat-treatment temperature increases, the ductility of PVDF increases, resulting in a higher elongation at break of the CNFMs. However, when the temperature rises above 160 °C, most of the PVDF begin to melt and the soft CNFMs become harder, causing a decrease of the elongation at break of the CNFMs. Therefore, the CNFMs with better mechanical properties could be prepared by controlling the heat-treatment temperature.

**Figure 14:**
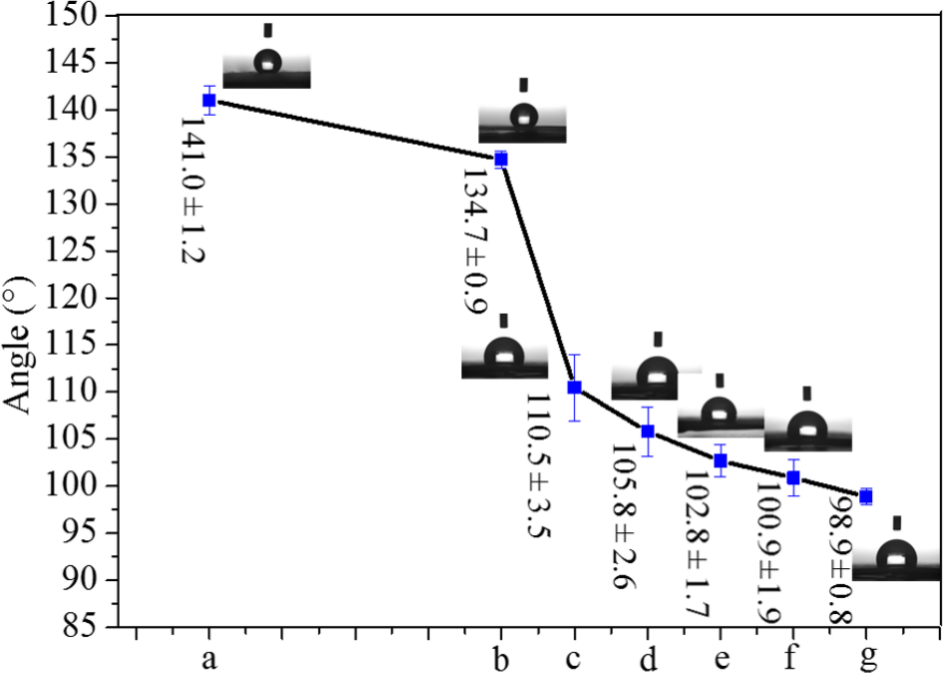
Contact angles of (a) the CNFMs without heat treatment and (b-g) the CNFMs with heat treatment for 2 hours ((b) 80°C; (c) 100°C; (d) 120°C; (e) 140°C; (f) 160°C; (g) 180°C).

**Wetting properties:** The CA values of the CNFMs obtained without and with heat treatment are illustrated in Figure 14. The hydrophobicity of the heat-treated CNFMs is smaller than that of the CNFMs without heat treatment, due to the formation of hydrophilic CuO and ZnO nanoparticles during the heat treatments. In addition, the hydrophobicity of the heat-treated CNFMs gradually decreases with the increase of the heat-treatment temperature, indicating that Cu(Ac)_2_ and Zn(Ac)_2_in the CNFMs are completely transformed into CuO and ZnO under the higher heat-treatment temperatures.

#### Effect of the heat-treatment time on the morphology of the CNFMs

**Figure 15:**
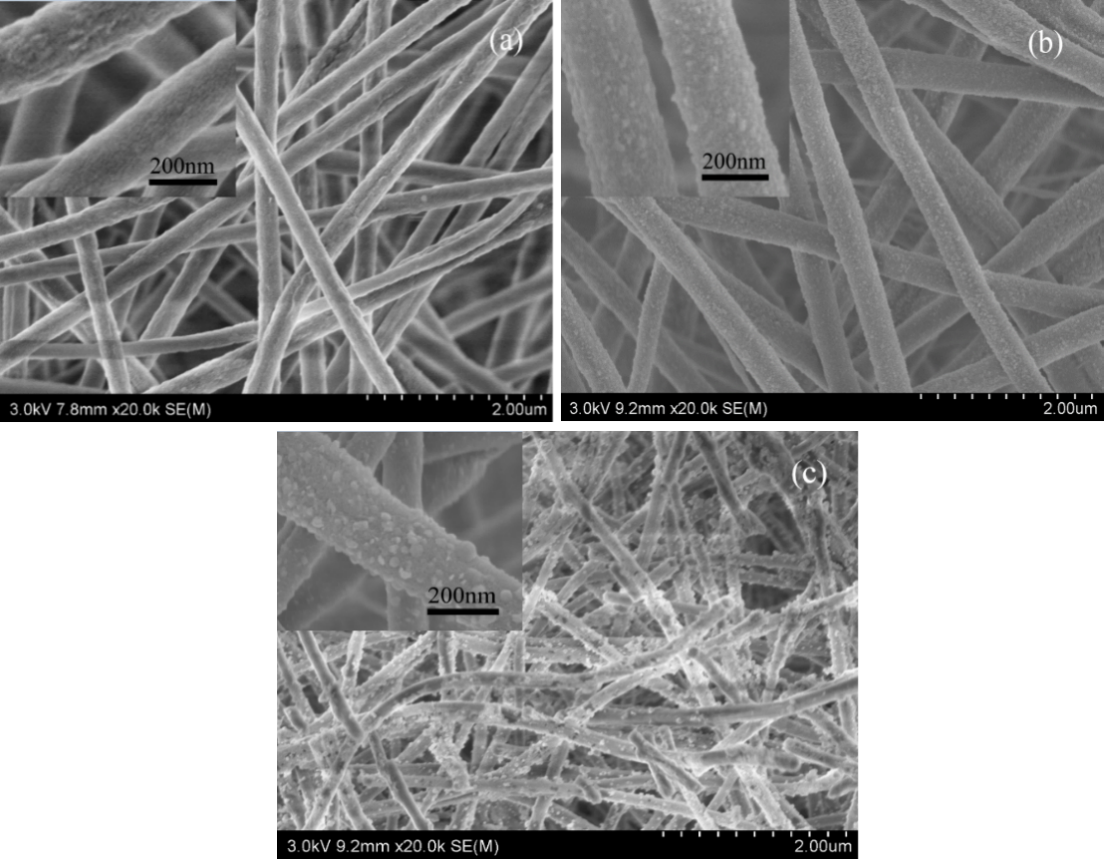
SEM images of PVDF/PAN CNFMs with CuO and ZnO nanoparticles obtained after heat treatment at 130 °C for different periods of time: (a) 2 h; (b) 8 h; (c) 16 h.

Figure 15 shows SEM images of PVDF/PAN CNFMs with CuO and ZnO nanoparticles obtained after heat treatment at 130 °C for 2, 6 and 18 h respectively. Longer heat-treatment times led to the formation of more CuO and ZnO nanoparticles. Therefore, a heat-treatment time of 18 h was selected for further experiments.

#### Characterization of heterostructured CuO–ZnO-loaded CNFMs

Based on the above PVDF/PAN CNFMs with CuO and ZnO nanoparticles obtained after heat treatment for 18 h at 140 °C, heterostructured CuO–ZnO-loaded CNFMs were fabricated using a hydrothermal method. The effects of growth solution concentration as well as temperature and time of the hydrothermal synthesis on the morphology of the heterostructured CuO–ZnO-loaded CNFMs were investigated by SEM. Also, structure and wetting properties of the CNFMs were studied.

#### Effect of the growth solution concentration on the morphology of the CNFMs

**Figure 16:**
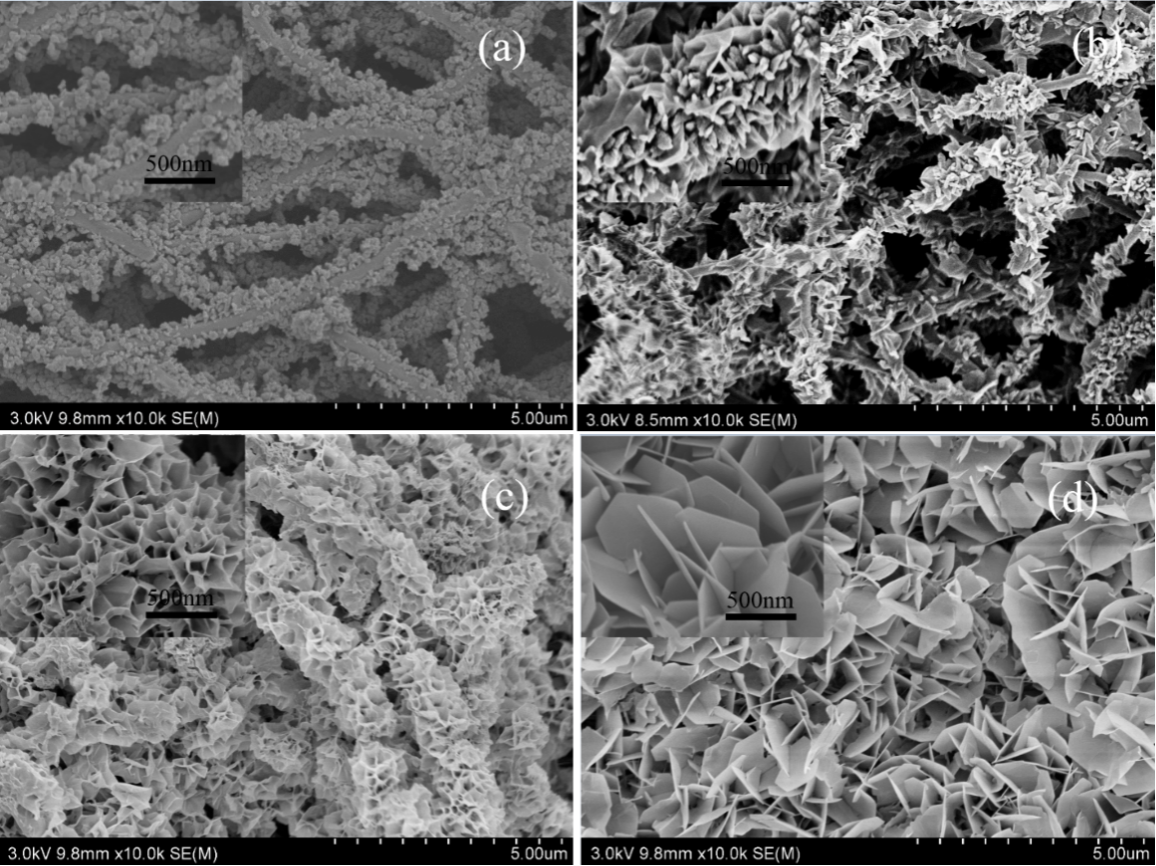
SEM images of heterostructured CuO–ZnO-loaded CNFMs obtained using different dilutions of the growth solution during hydrothermal synthesis: (a) 1:25; (b) 1:5; 1:2; (d) saturated solution.

Sizes and structure of CuO and ZnO nanocrystals varied gradually with the increase of the growth solution concentration during hydrothermal synthesis at 120 °C for 12 h (Figure 16). Higher concentrations of the growth solution led to the formation of more heterostructured CuO–ZnO. When the dilution is 1:25, CuO and ZnO crystals with irregular shape and size are deposited on the surface of the nanofibers and piled up disorderly (Figure 16a). When the dilution is 1:5, CuO and ZnO crystals are stacked on the surface of the nanofibers in granule-like or sheet-like structures with non-uniform distribution (Figure 16b). When the dilution is 1:2, the whole nanofiber surfaces are encapsulated by CuO and ZnO crystals, forming a heterostructure similar to a honeycomb (Figure 16c). When the saturated growth solution is used, CuO and ZnO crystals with regular polygonal sheet-like structures, similar to petals, are uniformly distributed on the whole nanofiber surfaces, leading to a very large specific surface area. Therefore, the saturated growth solution was used in the subsequent experiments.

#### Effect of the synthesis temperature on the morphology of the CNFMs

**Figure 17:**
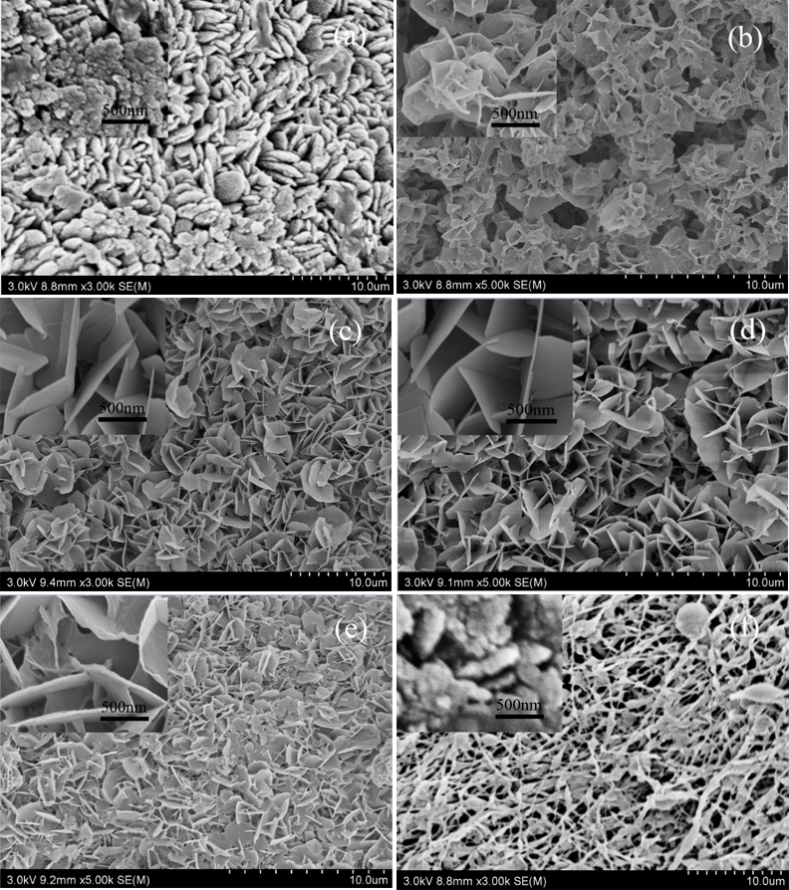
SEM images of heterostructured CuO–ZnO-loaded CNFMs after hydrothermal synthesis at different temperatures: (a) 80 °C; (b) 100 °C; (c) 120 °C; (d) 140 °C; (e) 160 °C; (f) 180 °C.

Figure 17 shows the SEM images of heterostructured CuO–ZnO-loaded CNFMs prepared using the saturated growth solution during hydrothermal synthesis for 12 h at different temperatures. When the hydrothermal temperature is 80 °C, the surface of the nanofibers is covered by a layer of thick pebble-like nanoparticles. When the hydrothermal temperature is 100 °C, the CuO–ZnO heterostructures similar to a honeycomb are obtained. When the hydrothermal temperatures are 120, 140 and 160 °C, the CuO–ZnO heterostructures formed were petal-like and resembled flowers. However, the heterostructures obtained at 120 and 140 °C are much more regular and thinner than those obtained at 160 °C. When the temperature reaches 180 °C, the nanofibers begin to shrink due to the melting of PVDF, and CuO–ZnO heterostructure do not form. Therefore, temperatures of 120–140 °C were chosen for hydrothermal synthesis in the subsequent experiments.

#### Effect of the time of hydrothermal synthesis on the morphology of the CNFMs

**Figure 18:**
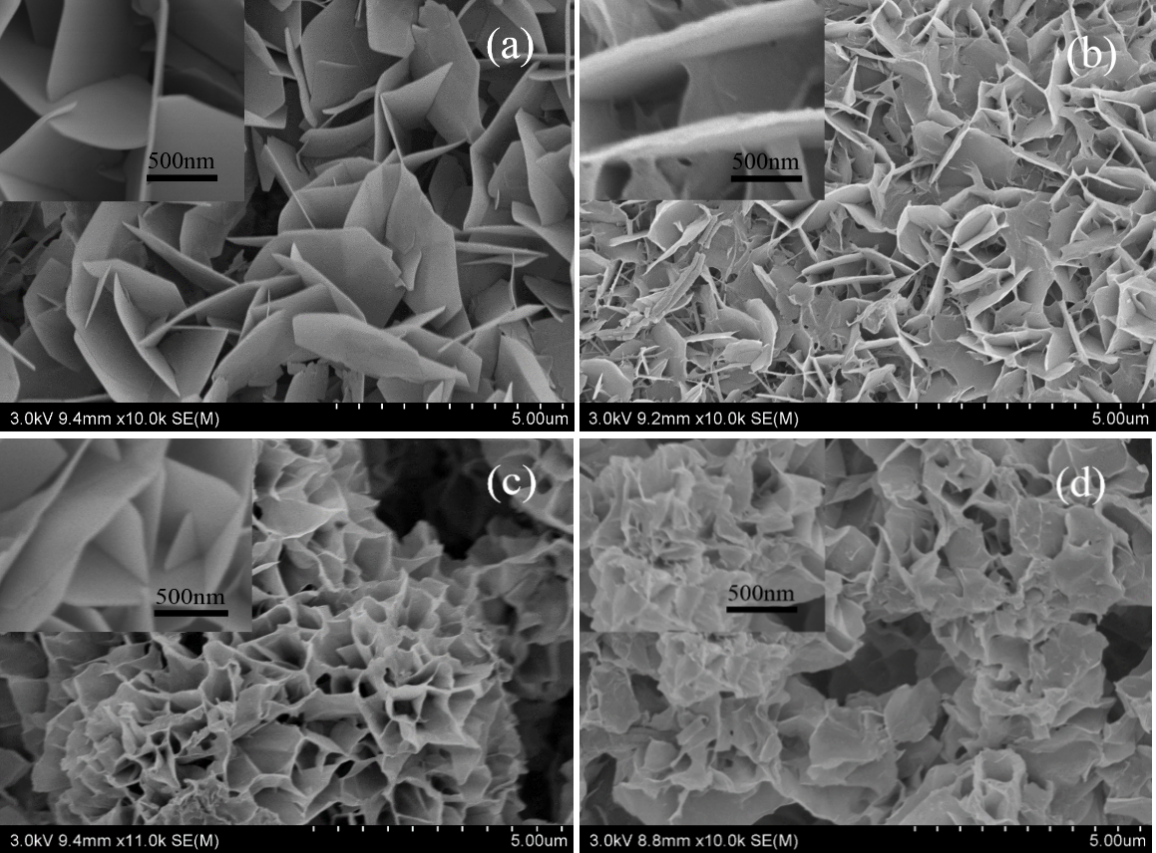
SEM images of heterostructured CuO–ZnO-loaded CNFMs after different times of hydrothermal synthesis: (a) 6 h; (b) 12 h; (c) 18 h; (d) 24 h.

Heterostructured CuO–ZnO-loaded CNFMs were fabricated using the saturated growth solution at 120 °C for different periods of times. The corresponding SEM images are shown in Figure 18. When the synthesis time is 6 h, the CuO–ZnO heterostructures are regularly stacked petal-like sheets with distinct edges and corners. After a synthesis time of 12 h, the CuO–ZnO heterostructures are also petal-like, but thicker than those after 6 h. When the synthesis time is 18 h, the CuO–ZnO heterostructure is similar to a honeycomb due to a continuous stacking of the sheets. After 24 h of hydrothermal synthesis, the CuO–ZnO heterostructures become disordered because a large number of sheets are formed. Hence, the hydrothermal synthesis time of 6 h was chosen for subsequent experiments.

#### XRD and EDS analysis of CuO–ZnO-loaded CNFMs

**Figure 19:**
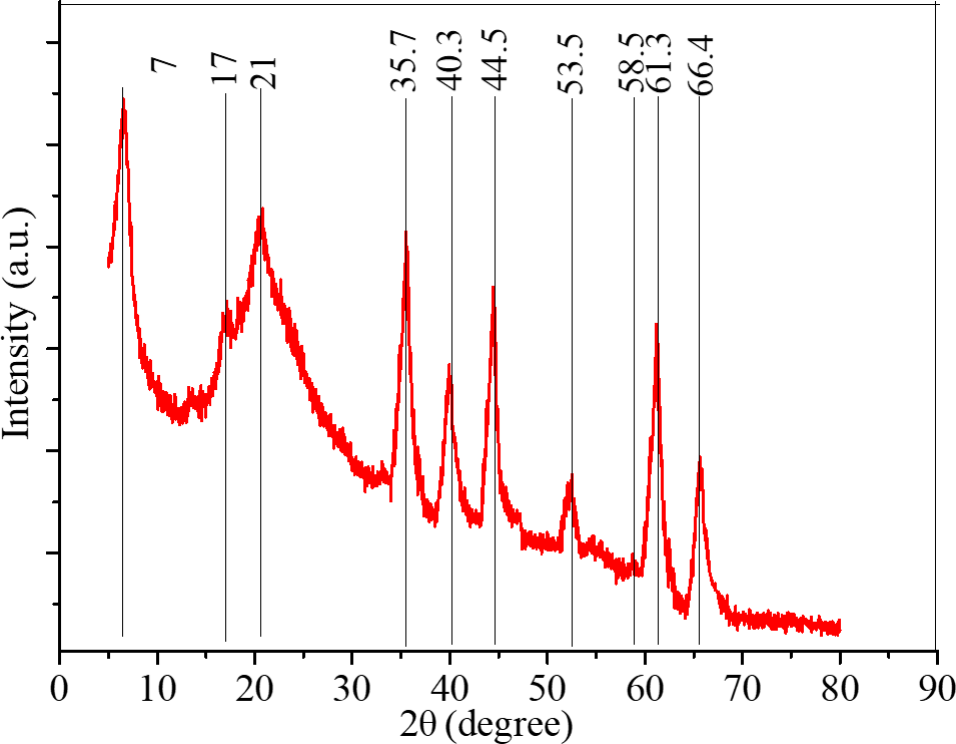
X-ray diffraction pattern of heterostructured CuO–ZnO-loaded CNFMs.

The X-ray diffraction spectrum of heterostructured CuO–ZnO-loaded CNFMs is given in Figure 19. The diffraction peaks of PAN and PVDF are located at 17° and 21°, respectively, and the superposition peak of Cu(Ac)_2_ and Zn(Ac)_2_ is at 7°, indicating remainders of Cu(Ac)_2_ and Zn(Ac)_2_ due to an incomplete decomposition during heat treatment. In addition, there is a strong diffraction peak at about 35.7° formed by CuO and ZnO together. The diffraction peaks at 44.5°and 66.4° belong to ZnO, while those at 40.3°, 58.5°, 53.5°and 61.3° belong to CuO. However, these peaks are slightly shifted due to the interaction of CuO and ZnO [43–45]. The XRD pattern show that CuO–ZnO heterostructures are formed on the surface of the nanofibers.

**Figure 20:**
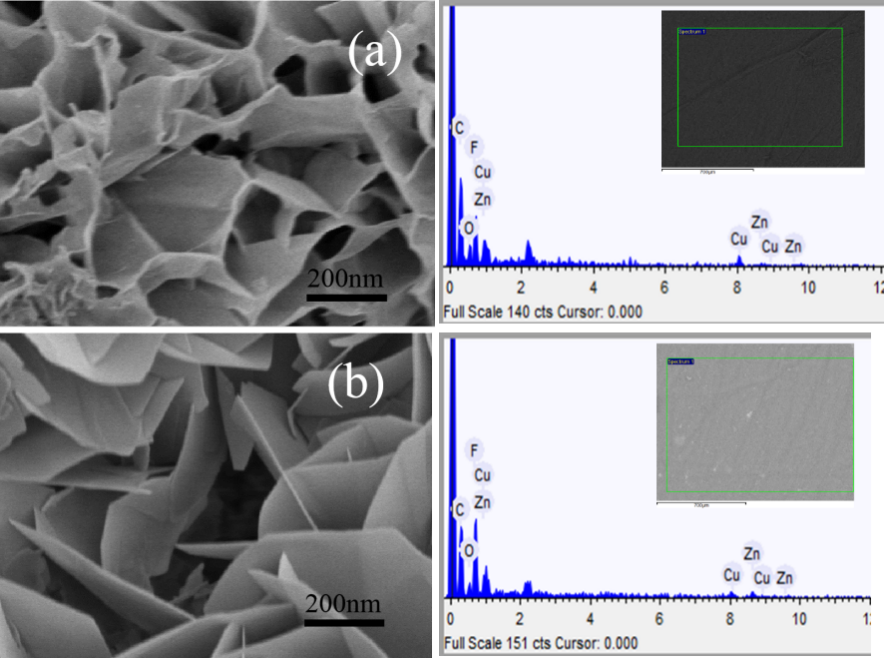
Elemental compositions of CNFMs with different CuO–ZnO heterostructures.

Figure 20 shows the EDS spectra of two different heterostructured CuO–ZnO-loaded CNFMs with different macroscopic brightness and microstructure. As given in Table 4, the content of CuO and ZnO sheets is higher in the sample in Figure 20a than in the sample in Figure 20b, resulting in a darker appearance of the CNFMs. In addition, in Figure 20a, the ratio between Cu and Zn is 3:2, which might be caused CuO crystals growing faster than ZnO crystals. In Figure 20b, the ratio between Cu and Zn is 1:1.

**Table 4:** Elemental composition of CNFMs with different CuO–ZnO heterostructures corresponding to Figure 20.

	element	wt %	atom %

Figure 20a	C	26.623	20.326
	H	5.937	54.383
	O	10.829	6.201
	N	11.220	7.337
	F	15.674	7.557
	Cu	17.908	2.501
	Zn	11.809	1.695

Figure 20b	C	35.110	23.515
	H	6.598	53.026
	O	4.561	2.290
	N	15.874	9.114
	F	24.630	10.415
	Cu	7.207	0.884
	Zn	6.020	0.756

#### Wetting properties of the CNFMs

**Figure 21:**
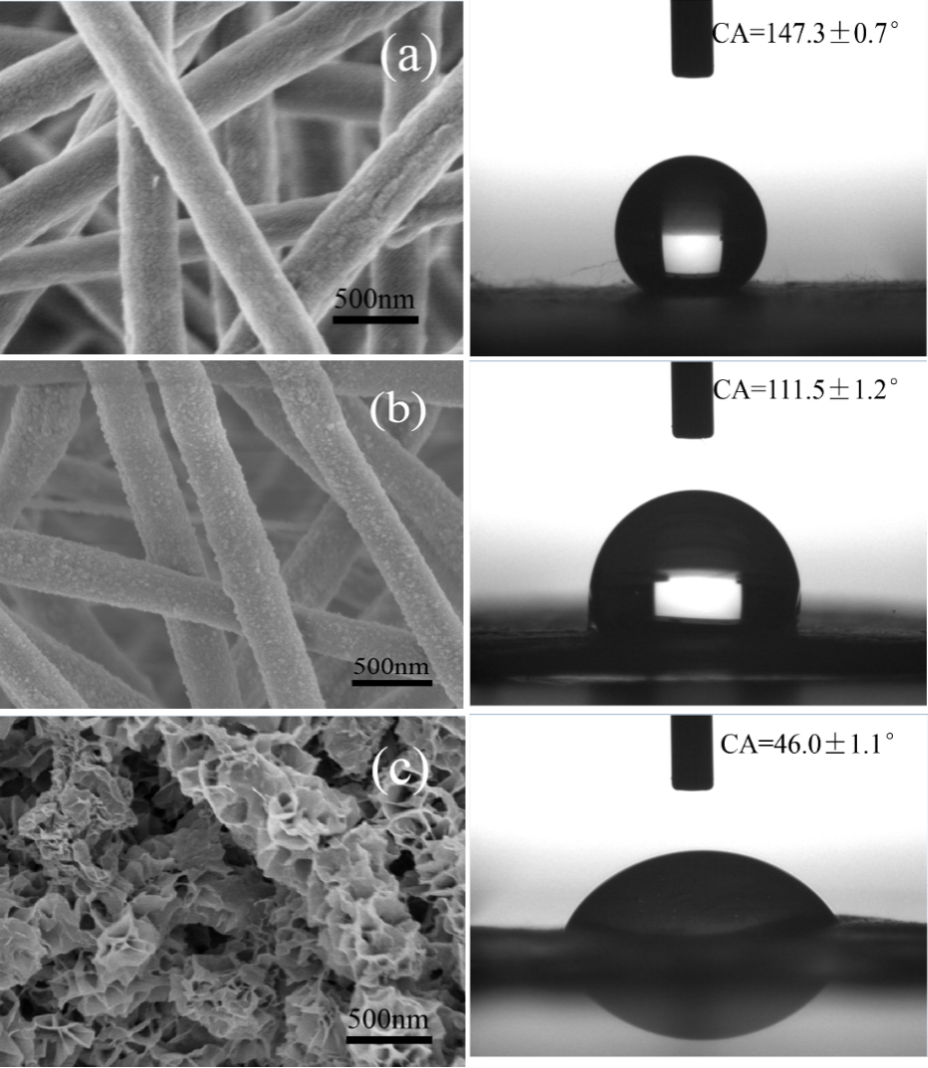
Morphologies and CA of CNFMs before and after heat treatment, and after hydrothermal growth: (a) without heat treatment; (b) with heat treatment; (c) with heat treatment and hydrothermal growth.

**Figure 22:**
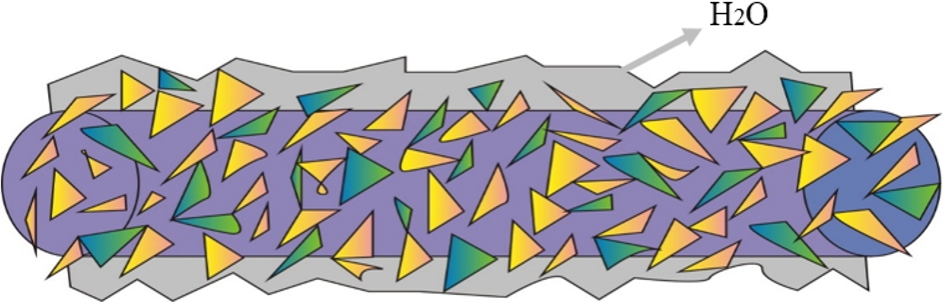
Mechanism of the hydrophilicity of CNFMs with CuO–ZnO heterostructures.

The influence of the CuO-ZnO heterostructures on the wettability of the CNFMs was investigated, and the morphologies and CA of CNFMs before and after heat treatment and hydrothermal growth are given in Figure 21. The results show that the wettability of the CNFMs changed from hydrophobicity to hydrophilicity after heat treatment and hydrothermal growth. The main reason is that hydrophilic CuO and ZnO grows on the surface of the nanofibers. The corresponding mechanism of hydrophilicity is illustrated in Figure 22. When hydrophilic CuO and ZnO are loaded on the surface of nanofibers, water droplets can spread into the gaps of the flaky CuO–ZnO structures, leading to the hydrophilic character of the CNFMs.

#### Photocatalytic degradation experiment

**Figure 23:**
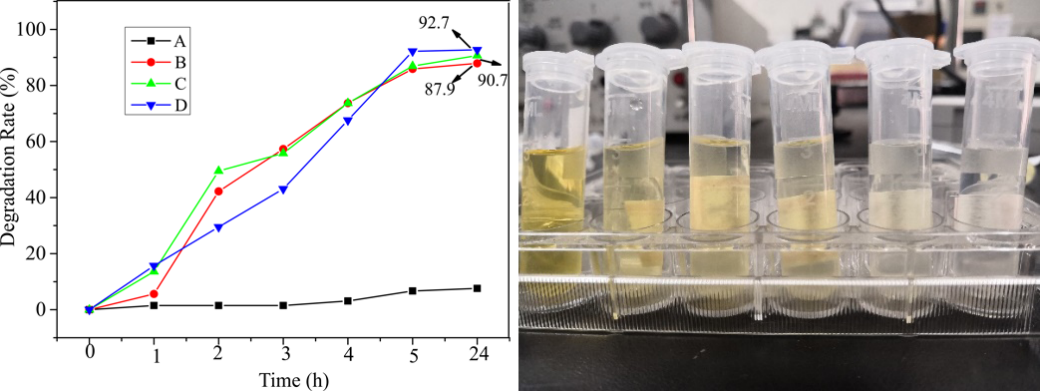
Left: degradation rate of methyl orange solutions using samples A, B, C and D as a function of the time. Right: degradation of methyl orange solutions after different periods of time using sample D.

Recently, researches on the application of CuO–ZnO nanocomposites in the photocatalytic degradation of organic pollutants were performed [46–48]. In this study, the photocatalytic degradation experiments under UV irradiation were carried out using the four prepared CNFMs in order to degrade a methyl orange solution with a concentration of 10 mg/L. The four CNFMs were electrospun PVDF/PAN CNFMs, CuO-loaded PVDF/PAN CNFMs, ZnO-loaded PVDF/PAN CNFMs and heterostructured CuO-ZnO–loaded PVDF/PAN CNFMs, named A, B, C and D, respectively. The measured absorbance as a function of the time in the degradation process is shown in Table 5 and Figure 23. The degradation rate of methyl orange, defined as η, was calculated by the following formula:


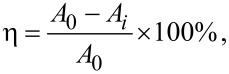


where *A*_0_ is the initial absorption of the solution and *A*_i_ is the currently measured absorption.

**Table 5:** Absorbance and degradation rate of methyl orange solutions functions of the time using samples A, B, C and D.

time (h)		0	1	2	3	4	5	24

absorbance	A	0.396	0.390	0.390	0.390	0.384	0.370	0.366
	B	0.396	0.374	0.229	0.169	0.107	0.056	0.048
	C	0.396	0.342	0.20	0.175	0.104	0.052	0.037
	D	0.396	0.334	0.279	0.154	0.086	0.032	0.029

degradation rate (%)	A	0	1.5	1.5	1.5	3.1	6.7	7.6
	B	0	5.6	42.2	57.3	73.7	85.9	87.9
	C	0	13.6	49.5	55.8	73.7	86.9	90.7
	D	0	15.7	29.5	43.1	67.6	92.2	92.7

Sample A showed almost no photocatalytic degradation of methyl orange, and the final degradation rate of 6.7% was likely due to the adsorption of a small amount of methyl orange on the CNFMs. Samples B, C and D all had a good photocatalytic degradation effect on methyl orange. The photocatalytic degradation rate of the sample D almost reached its maximum after 5 h. Compared with the final degradation rate of sample B and sample C, that of sample D was the highest with up to 92.7%. That meant the heterostructured CuO–ZnO-loaded CNFMs had the best photocatalytic degradation effect due to the wider UV adsorption region.

**Figure 24:**
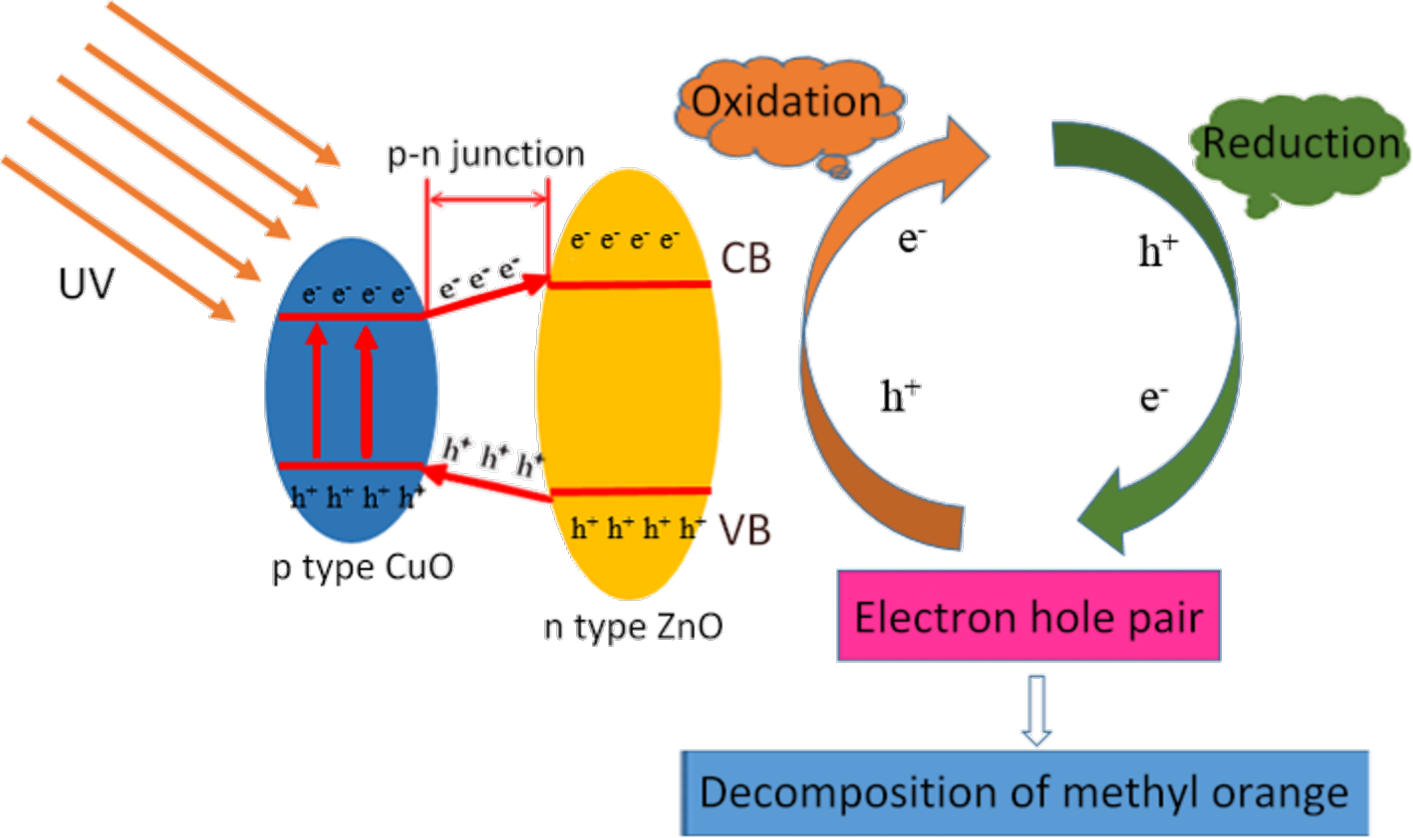
Mechanism of the photocatalytic degradation of methyl orange using the heterostructured CuO–ZnO-loaded CNFMs.

The mechanism of photocatalytic degradation of methyl orange using the heterostructured CuO-ZnO-loaded CNFMs is illustrated in Figure 24. When the UV light irradiates the CuO-ZnO heterostructure on the CNFMs, the valence-band (VB) electrons are excited to the conduction band (CB), resulting in the formation of electron–hole pairs. Holes and electrons both act in the photocatalytic degradation of methyl orange. In addition, a large number of p–n heterojunctions are formed due to the combination of p-type CuO and n-type ZnO. Under UV irradiation, the excited electrons in the CB of CuO can easily move to the CB of ZnO, whereas the holes in the VB of ZnO can migrate into the VB of CuO. Thus, the number of photoexcited holes in the CuO–ZnO heterostructure increases [49], which leads to the higher photocatalytic activity.

The results of three consecutive photocatalytic degradation experiments using the same sample D are given in Table 6. The degradation rates decrease, but remains above 90%. This means that the heterostrutured CuO–ZnO-loaded CNFMs can be reused to degrade methyl orange for at least three times.

**Table 6:** Degradation rates during consecutive photocatalytic degradation experiments using the sample D.

number of experiment	absorbance		degradation rate (%)
	0 h	5 h	

1	0.396	0.029	92.2
2	0.396	0.033	91.4
3	0.396	0.039	90.1

## Conclusion

Heterostructured CuO–ZnO-loaded CNFMs were prepared by a combination of electrospinning technology, heat treatment and hydrothermal synthesis. The effects of electrospinning, heat treatment and hydrothermal parameters on morphology, structure and properties of the CNFMs were investigated, and the optimal process parameters were determined. The results showed that when Cu(Ac)_2_/Zn(Ac)_2_ = 1:1, PVDF/PAN = 5:5 and [Cu(Ac)_2_/Zn(Ac)_2_]/[PVDF/PAN] = 1:2, morphology and mechanical properties of the electrospun CNFMs were optimal and the Cu(Ac)_2_ and Zn(Ac)_2_ nanoparticles were evenly distributed on the surface of the nanofibers. When the heat-treatment temperature was in the range of 120–140°C and the duration of heat treatment was 18 h, morphology, structure and mechanical properties of CuO and ZnO-loaded CNFMs were optimal. When the growth solution was saturated, the hydrothermal temperature was 120–140 °C and the duration of the hydrothermal synthesis was 6 h, morphology and structure of heterostructured CuO–ZnO on the CNFMs were the best.

Then, the heterostructured CuO–ZnO-loaded CNFMs prepared using the optimal process parameters were applied in the photocatalytic degradation of methyl orange, and the mechanism of photocatalytic degradation was investigated. The photocatalytic degradation of methyl orange was carried out for 24 h, and the final degradation rate was 92.7%, which was higher than that of CNFMs with CuO or ZnO only. In addition, the heterostructured CuO–ZnO-loaded CNFMs could be reused to degrade methyl orange for at least three times, and the degradation rate remained above 90%.
